# The cytoskeleton of pathogenic protists

**DOI:** 10.1042/BSR20254047

**Published:** 2026-04-10

**Authors:** Wanderley de Souza, Ana Paula Gadelha, Marlene Benchimol

**Affiliations:** 1 Laboratório de Ultraestrutura Celular Hertha Meyer, Centro de Pesquisa em Medicina de Precisão, Instituto de Biofísica Carlos Chagas Filho, Brazil; 2 Centro Nacional de Bioimagem e Biologia Estrutural, Universidade Federal do Rio de Janeiro, Brazil; 3 Unigranrio-Universidade do Grande Rio-Duque de Caxias-Rio de Janeiro, Brazil; 4 Instituto Nacional de Metrologia, Qualidade Industrial e Tecnologia, Colombia

**Keywords:** Apicomplexa, Cytoskeleton, Entamoeba histolytica, Giardia intestinalis, Protists, Trichomonadidae, Trypanosomatidae

## Abstract

The cytoskeleton is a key component of eukaryotic cells, including protists. It is composed of microtubules, microfilaments, and intermediate filaments. Here, we review the available information on the cytoskeleton of several relevant pathogenic protists, including Trypanosomatidae, Apicomplexa, Trichomonadidae, *Giardia intestinalis*, and *Entamoeba histolytica*. In protists, the first two components, made of tubulins and actin, predominate. Usually, they associate with each other and with other components to form complex structures. Emphasis is given to the following structures: flagellum, flagellar-cell body adhesion zone, paraflagellar rod, sub-pellicular microtubules, cytostome, conoid, adhesive disc, funis, median body, costa, axostyle, parabasal filaments, and clockwise filaments. On the other hand, filamentous structures made of not yet completely characterized proteins form structures such as the costa. Each structure is analyzed using morphological information obtained through modern microscopy techniques and biochemical data.

## Introduction

The term cytoskeleton initially suggests a rigid structural organization that maintains the cell’s shape. Today, however, we know that it corresponds to a highly dynamic structural organization, both in terms of a state of organization that allows for sharp and often rapid changes in the shape of the cell but also a dynamic intracellular state that regulates the movements of macromolecular complexes and organelles in the cell cytoplasm, as well as intranuclear macromolecular complexes. The cytoskeleton is formed and regulated by dozens of proteins. However, from a structural perspective, most eukaryotic cells comprise three main components: actin filaments (microfilaments), intermediate filaments, and microtubules. Several excellent reviews on these structures in mammalian cells are available [1–5]. In this review, we will concentrate on the most relevant information on the cytoskeleton of parasitic protists. Given the limited space, we selected a few of the most relevant pathogenic protists for humans. Each selected one presents a distinct cytoskeletal organization. Therefore, they are described separately. It is not possible to compare them uniformly.

## The cytoskeleton of trypanosomatids

**Figure 1:**
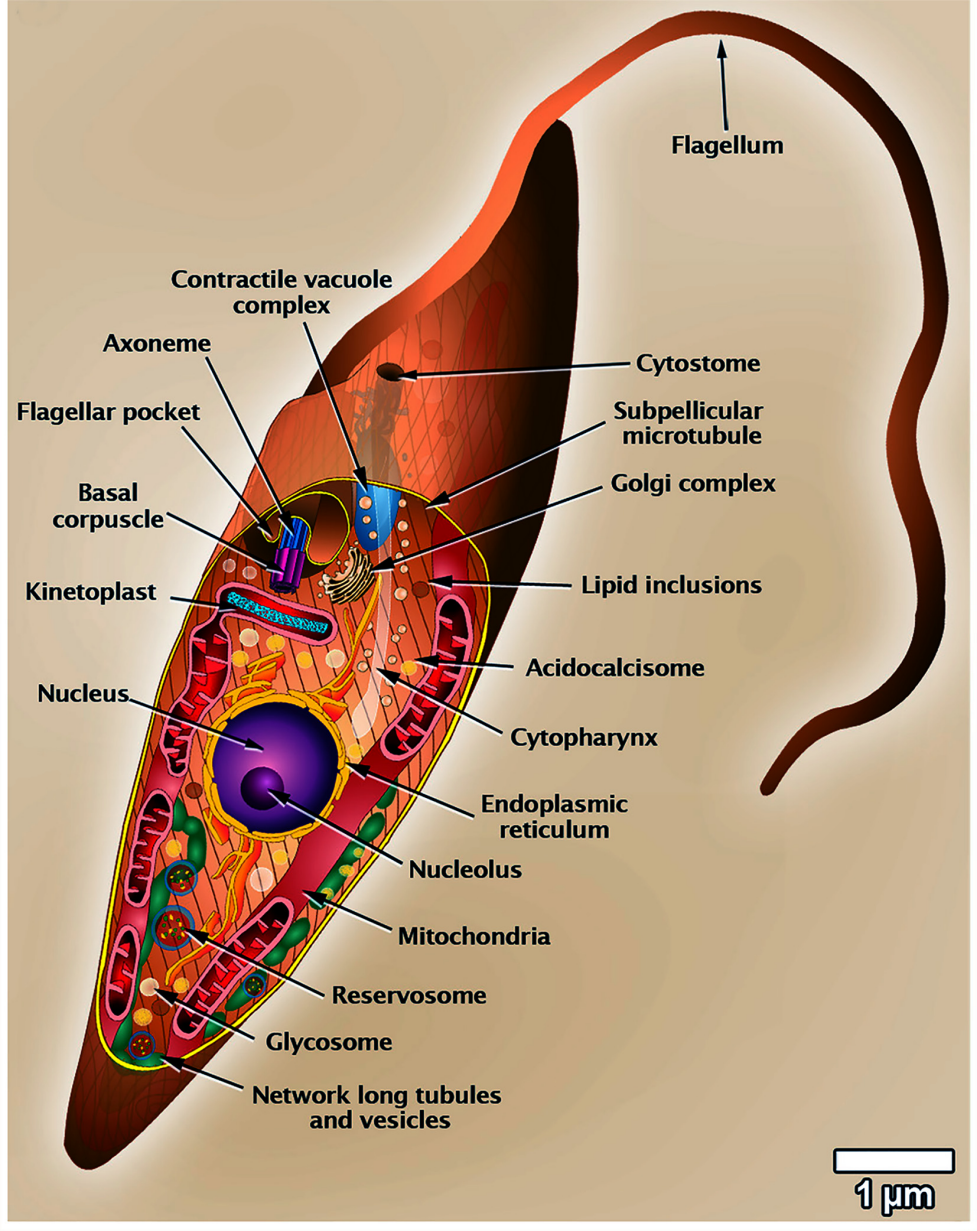
*Trypanosoma cruzi* scheme Representation of a longitudinal section of an epimastigote form of *T. cruzi*, where the most relevant structures and organelles are indicated. This scheme was developed by two of the authors [6].

The Trypanosomatidae family comprises many species that are agents of parasitic diseases in humans, animals of veterinary interest, wild animals, and even plants (*Phytomonas*). There are several reviews dealing with *Trypanosoma brucei*,* Trypanosoma cruzi*, and *Leishmania*. Figure 1 illustrates a general scheme of *Trypanosoma cruzi* organization [6]. Below, we will describe the relevant cytoskeleton structures found in trypanosomatids.

## The flagellum

The flagellum is a characteristic morphological feature of all members of the Trypanosomatidae family. There is only one flagellum per interphasic cell. The length of the flagellum of *T. cruzi* varies according to the developmental stage. It is very short in amastigotes, proliferating within the cell. Its length increases during transformation from amastigotes to promastigotes, epimastigotes, and trypomastigotes. In these last two stages, it is always attached to the protozoan body, ending freely in the anterior portion. It always emerges from the flagellar pocket originating from basal bodies, located just in front of the kinetoplast, and is made of an axoneme of dynein-bound doublet microtubules (DMTs). It presents a specialized region at its tip, known as the flagellar cap. In the case of *T. brucei*, there is also a specialized structure in the new flagellum formed during cell division that is laterally connected to the old flagellum. This structure is known as a connector. A significant number of proteins have been found in the flagellar tip and the connector of *T. brucei* [7].

## The flagellar-cell body adhesion

In all trypanosomatids, the flagellum attaches to a portion of the cell body. In developmental stages such as the epimastigote and trypomastigote, this adhesion is extensive. On the protozoan cytoplasmic side, the presence of an electron-dense material is evident. It is enhanced in samples prepared with ethanolic phosphotungstic acid, which presumably reveals the presence of basic proteins [8]. This complex, designated the Flagellum Attachment Zone (FAZ), has been characterized in detail in *Trypanosoma brucei*. The ability to knock down specific proteins in parasites using Interference RNA (RNAi) has enabled further dissection of the Flagellum Attachment Zone (FAZ) [9]. The interaction between the FLA-1 and FLA1-BP proteins in *T. brucei* is one of the most well-characterized aspects of the structural organization of the flagellar adhesion zone (FAZ). This interaction is crucial for anchoring the flagellum to the parasite’s cell body and plays a central role in FAZ assembly throughout the life cycle. Evidence suggests that FLA-1 and FLA1-BP possess transmembrane and extracellular domains, with interaction primarily occurring through their extracellular regions [10–12]. More recently, the FAZ of *T. cruzi* was characterized. Several proteins were found associated with the flagellum, the link of the flagellum to the cell body, the membrane of the cell body, as well as cytoplasmic structures facing the cell body membrane. It was also shown that interference with several proteins leads to detachment of the flagellum from the cell body, followed by changes in the general shape of the protozoan [13]. In *T. cruzi*, TcGP72 and TcFLA-1BP proteins are associated with distinct regions of the FAZ: TcGP72 is localized in the FAZ domain of the flagellum, whereas TcFLA-1BP is in the FAZ domain of the cell body [14]. The FLA1 protein in *T. brucei* and *Leishmania* exhibits homology to TcGP72 in *T. cruzi* [9,15]. Previous studies have demonstrated that deletion of these proteins alters cell growth, cytokinesis, and parasite morphometry, with more pronounced effects observed in the TcGP72−/− mutant. Although the absence of FLA-1 or FLA1-BP in both parasites leads to morphologically aberrant forms, the complete disruption of the FAZ observed in the TcGP72−/− mutant directly affects metacyclogenesis and intracellular organelle rearrangement. Its knockdown results in detachment of the flagellum from the cell body, without significant changes in the protozoan’s shape or its ability to divide. In the case of *T. brucei*, the equivalent protein designated as FLA1 also contains NHL repeats. It is part of a complex that also contains a predicted glycosylated binding protein (FLA1BP). FLA1 seems to be in the membrane lining the cell body, while FLA1BP is in the flagellar membrane at the FAZ domain. In analogy to what happens with desmosomes, it was suggested that these proteins are analogous to cadherins.

## The axoneme

**Figure 2:**
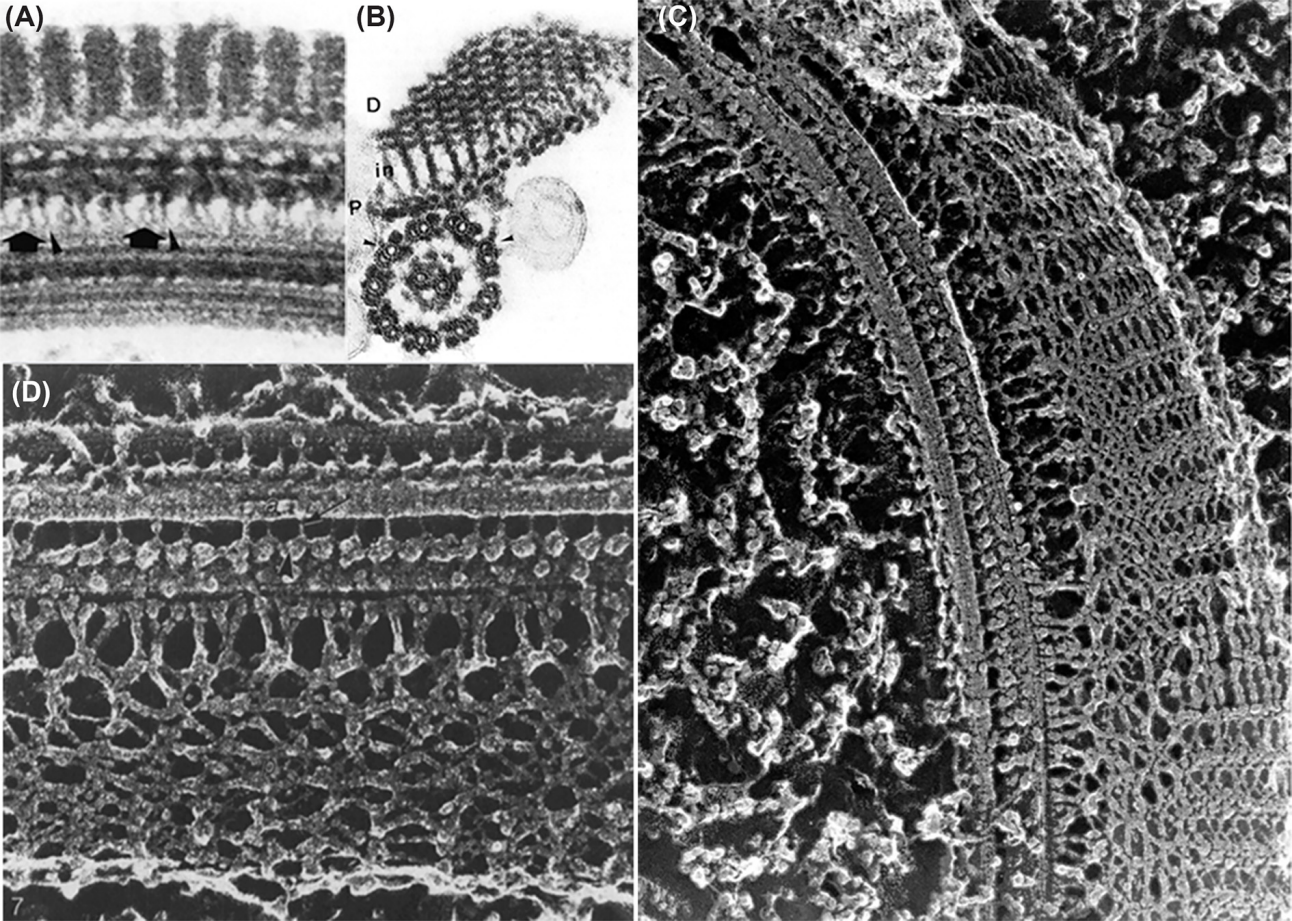
Details of the flagellum axoneme and the PFR of trypanosomatids (**A** and **B**) thin sections of flagella fixed in glutaraldehyde-tannic acid [16]. (**C** and **D**) Freeze-fracture-deep-etching replicas showing the components of the flagellar axoneme, dynein arms, and the components of the PFR [17]. All images are from one of the author’s articles (WS).

The axoneme originates from the basal body that is located at the base of the flagellum and close to the kinetoplast. There are filamentous connections between the basal body and the kinetoplast. The basal body is formed by nine microtubule triplets containing tubulin, including γ and ε-tubulin. Each triplet will originate one doublet of the axoneme. At the transition zone between the basal body and the axoneme, there are nine peripheral microtubule doublets without the central pair. The axoneme contains one central pair of microtubules that are connected to 9 pairs of peripheral microtubule doublets (Figure 2A and B) [16]. Each doublet presents two dynein arms (outer and inner dynein arms). These arms connect adjacent microtubule doublets. There are radial spokes that connect the peripheral microtubules to the central pair and the nexin–dynein regulatory complex, which, in some way, regulates the function of dyneins, allowing their sliding and generating motility.

Recent cryo-electron microscopy studies of the flagellum in trypanosomatids provide new and relevant insights. Studies on *Crithidia and Leishmania* revealed 27 trypanosomatid-specific microtubule inner proteins, a specialized dynein-docking complex, and the presence of paralogous proteins that enable high-order periodicities or proximal-distal patterning [18]. Studies of *T. brucei* identified 154 axonemal proteins, both inside and outside the DMTs, and, together with genetic and proteomic analyses, revealed the conserved and trypanosome-specific foundations of flagellum assembly and motility. A comparative analysis of atomic models from pre- and post-power-stroke states revealed that dynein structural changes drive the sliding of adjacent DMTs during flagellar beating [19]. The flagellum grows and is maintained by the transport of intraflagellar particles that migrate from the base to the tip and vice versa, with the participation of the motor kinesins and dyneins [19–22]. In trypanosomes, all IFT (intraflagellar transport) genes are conserved and essential for flagellum elongation. At the base of the flagellum are concentrated the IFT proteins, and IFT trains are found along microtubule doublets. According to [23], parasites with knockdown of IFT kinesin, achieved through RNAi, exhibited shorter flagella when the IFT train frequency was reduced. The decrease in tubulin transport could also prevent flagellum elongation. Cryoelectron microscopy has recently been used to analyze IFT organization in detail in *T. brucei* [24]. The tip of the flagellum contains a complex of many proteins that form a structure known as the cap. In addition, in the case of *T. brucei*, there is another protein organization in the tip of the new flagellum of dividing cells that connects the tip of the new and short flagellum to the lateral side of the old and longer flagellum [7].

## The paraflagellar rod

The paraflagellar rod (PFR) is a structure found in most trypanosomatids and located at the side of the flagellar axoneme to which it is connected (Figure 2C and D). It is composed of a complex network of filaments of varying thickness, and at least three distinct domains can be identified. The proximal one is formed by two plates connected to the axoneme doublets 4 to 7 by short filaments. Plate-like filamentous structures also form the intermediate domain. The distal portion is formed by several plates (the number varies among trypanosomatid species) [17,25–27]. Several proteins are found in the PFR. The major ones are designated PFR 1 and 2, with molecular weights of around 70 kDa. PFR1 is found in all trypanosomatids, even in those species containing an endosymbiont, and that do not have a complete PFR. *Angomonas deanei* PFR2 is crucial for the assembly of the complete PFR [28]. An induced PFR2 (snl-1) mutant exhibited changes in shape and motility, and even a loss of infectivity in animals [25,26]. More detailed biochemical analysis, including proteomics, indicates the presence of approximately 30 proteins in the PFR. Some, such as adenylate kinase, PI3 kinase, and calmodulin, may be involved in cell signaling. For instance, the silencing of calmodulin interferes with the assembly of the PFR and flagellar motility [29].

## Cytoplasmic microtubules

**Figure 3:**
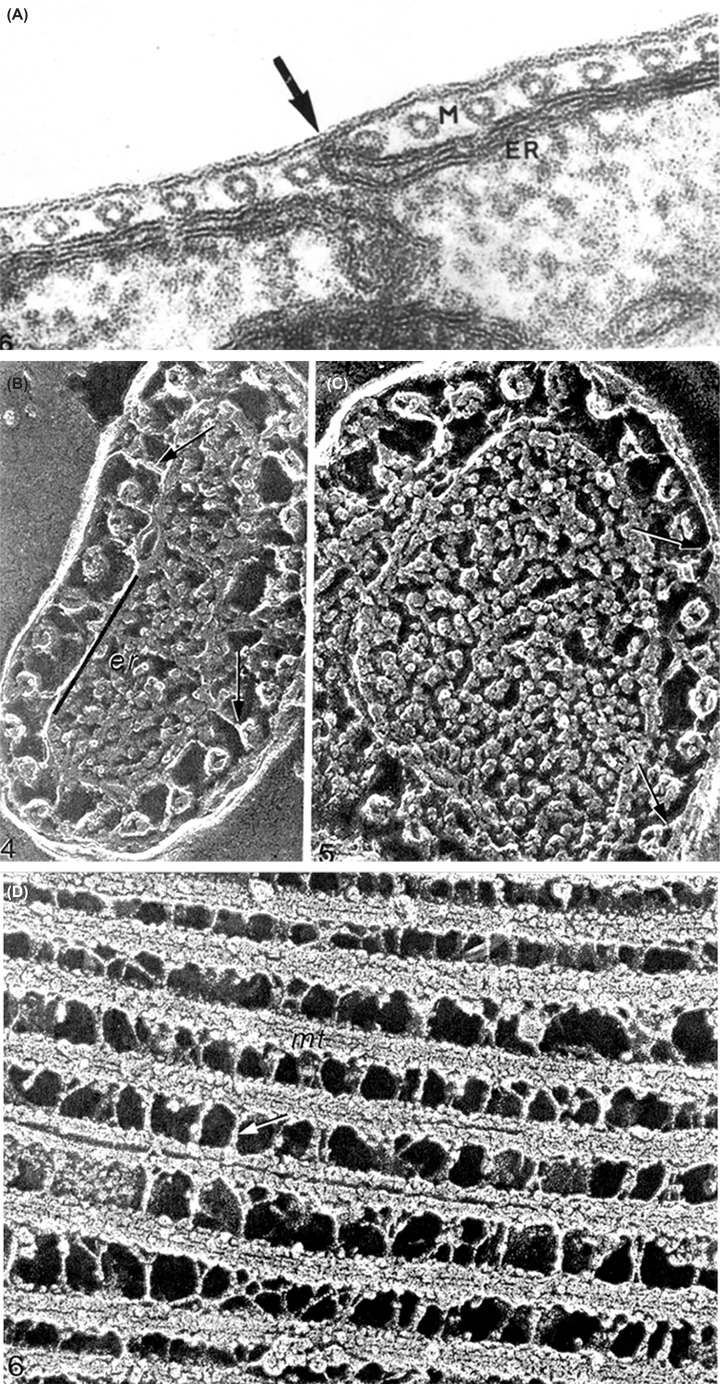
Microtubule details of trypanosomatis Images of thin section (**A**) and freeze-fracture deep-etching replicas (**B–D**) show the sub-pellicular microtubules (arrow) and their association with the plasma membrane, as well as connections between them and with the plasma membrane. (A), after [30]; (B–D) after [17]. All images are from one of the authors (WS).

**Figure 4:**
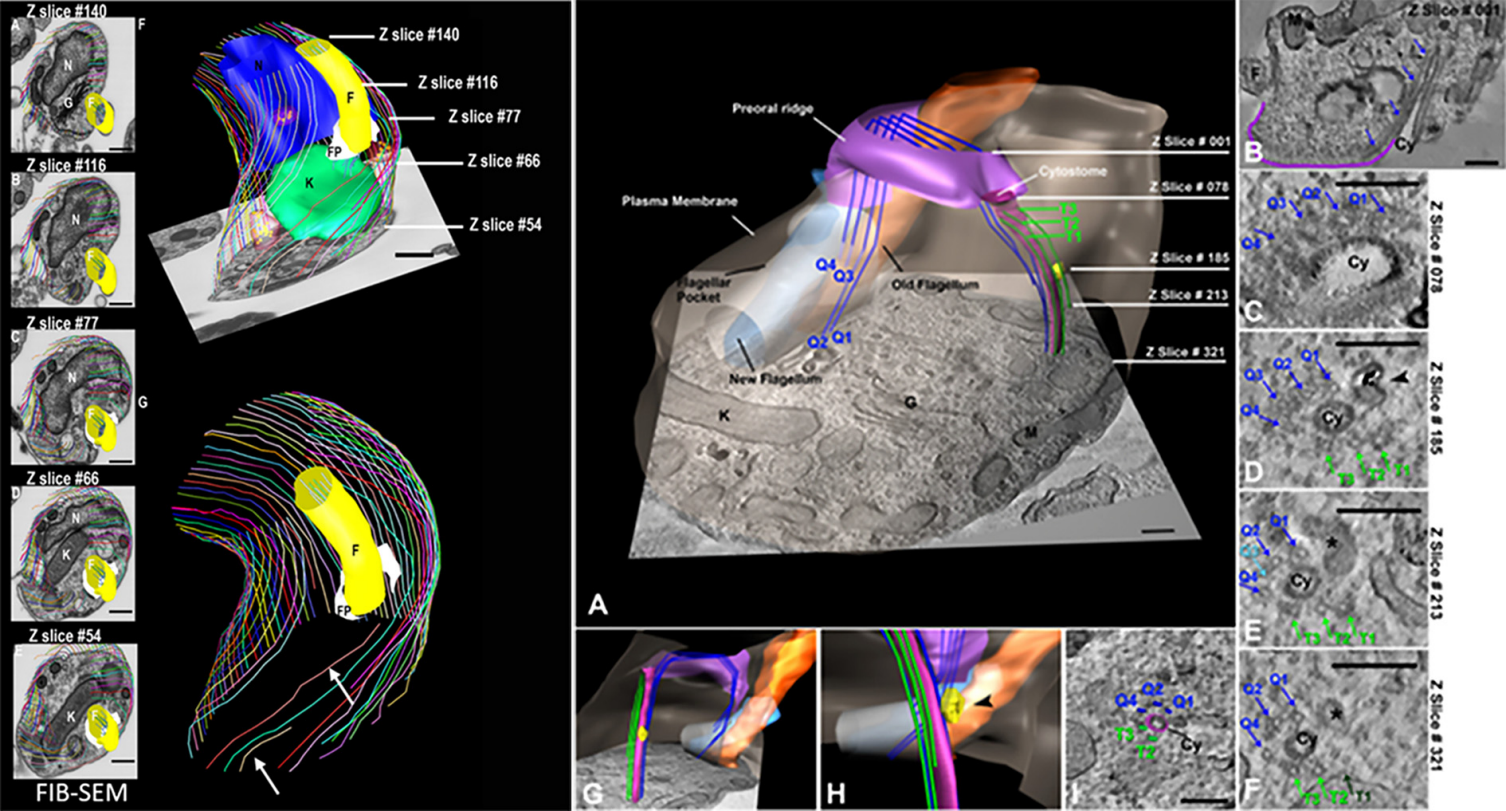
Images and model of the anterior region of a *T. cruzi* epimastigote Left (**A–E**): A sequence of FIB**–**SEM images from the central region of the cell. (**F**) A 3D model of the cell is shown in (A**–**E). The model shows the anterior region of a *T. cruzi* epimastigote cell. Near the flagellar pocket region (the portion lacking SPMT), it was possible to observe microtubules ending more abruptly. (**G**) Nucleus (N), blue; kinetoplast (K), green; flagellum (F), yellow; flagellar pocket (FP), white. Bars: 200 nm. The schematic drawing delineates the region analyzed in the 3D model. SPMTs, subpellicular microtubules (after [38]). Right: The distribution of microtubules along the cytopharynx. Serial tomograms show the positioning of microtubules along the cytopharynx. (A) View of the 3D model showing the beginning of the microtubule quartet (Q1 to Q4, blue) that follows the path of the preoral ridge (purple) and then bends into the cytoplasm, accompanying the cytostome cytopharynx (pink). A microtubule triplet (T1 to T3, green) originates from a position adjacent to the opening of the cytostome and accompanies the cytopharynx. (B–F) Virtual slices of the tomogram representing the regions indicated in (A). (B) The microtubule quartet (blue arrows) can be seen lining the cytopharynx (Cy) and the preoral ridge (purple contour). In (D), we can see the full set of microtubules around the cytopharynx and a vesicle (black arrowhead) containing Tf-Au particles (black dots) close to the naked side of the cytopharynx. This vesicle is also shown in yellow in the models in (**G** and **H**). Other membrane-bound compartments were observed close to the naked side of the cytopharynx (asterisks in E and F). As the cytopharynx becomes thinner and extends deeper into the cell, microtubules Q3 from the quartet (E) and T1 from the triplet (F) disappear. The light blue arrow and the dark green arrow in (E) and (F), respectively, indicate the very end of these microtubules. (**G**) Back view of the model in (A), in which the positioning of microtubules at the flagellar pocket, preoral ridge, and the cytostome can be better visualized. (**I**) At the most distal portion of the cytopharynx (relative to the cytostome), only five microtubules were seen around this structure, arranged in a half-moon shape. K, kinetoplast; M, mitochondrion; G, Golgi complex; F, flagellum; orange, new flagellum; light blue, old flagellum. Scale bars: 200 nm. (After [37]). All images are from one of the authors (WS).

One characteristic feature of trypanosomatids is the presence of cytoplasmic microtubules. Among them, the so-called subpellicular microtubules (SPMTs) are the most abundant (Figure 3). They correspond to a network of tubules formed by a longitudinal array of α- and β-tubulin heterodimers forming a helical pattern along the long axis of the protozoan body (Figures 3 and 4). These microtubules originate at the anterior region and project toward the posterior region. Their plus ends are in the posterior region. They are located beneath the plasma membrane and are spaced at a constant distance from each other. Filamentous bridges connect the microtubules to the plasma membrane (Figure 3). The number of these bridges varies according to the cell region [30].

The biogenesis of microtubules was followed using the YL1/2 monoclonal antibody, which binds to the tyrosinated C-terminal end of α-tubulin. A carboxypeptidase may remove the tyrosine upon the incorporation of α/β-tubulin dimers into the microtubule [31,32]. Using a tetracycline-inducible YFP-α tubulin expression system, it was shown that in *T. brucei* procyclic forms, new microtubule synthesis occurs during the cell cycle, mainly in the region between the old and the new flagellum formed during cell division. The available information suggests that during cell division, when the cell diameter increases, intercalation and posterior extension of new microtubules occur. Tubulin modifications, such as acetylation and detyrosination, have been analyzed in trypanosomatids. They have a high proportion of acetylated alpha tubulin, especially in the sub-pellicular microtubules, axonemal microtubules, and mitotic microtubules [33,34]. Acetylation of lysine 40 on α-tubulin is a conserved process. It was shown that overexpression of tubulin acetyltransferase induced morphological changes, including impairment of kinetoplast division [35]. Tubulin detyrosination was further analyzed in *Leishmania*, where its absence was shown to significantly disrupt microtubule dynamics and cytoskeletal remodeling during the transition from promastigotes to amastigotes [35]. One special feature of these microtubules is their remarkable stability, which allows them to remain intact throughout the cell cycle. In addition, they are quite resistant to physical and chemical treatments that typically depolymerize microtubules, such as low temperatures, high pressure, and incubation with compounds like colchicine. It has been shown that Taxol, a microtubule-stabilizing agent, prevents completion of the *T. cruzi* cell cycle [36]. In addition to the sub-pellicular microtubule, there are at least three other cytoplasmic microtubule sets. The first one recognized is the microtubule quartet that originates at the basal body and wraps around the flagellar pocket, intercalating into the sub-pellicular microtubule array at the point where the flagellum exits the pocket and lies adjacent to the FAZ filament [37]. In *Leishmania*, the quartet microtubules grow toward the anterior region. A second group, also composed of four microtubules, is found in species that present a cytostome–cytopharinx complex, as in *T. cruzi*. They originate in the flagellar pocket area underlying the preoral region, reach the cytostome, and follow the cytopharynx until its extremity. The third group consists of three microtubules that originate near the cytostome and follow the cytopharinx (Figure 4) [38].

It is important to note that the trypanosomatid genome encodes several tubulin isoforms, including α, β, γ, and ε, which are still poorly characterized. As in other microtubules, several proteins have been identified as interacting with the sub-pellicular microtubules and are considered microtubule-associated proteins (MAPs). Some of these proteins, such as those of 52 and 33 kDa, can form cross-links on mammalian microtubules. Others, such as TbAIR9, are found throughout the microtubule, whereas others, such as CAP15 and CAP17, localize mainly to the anterior portion of the microtubule network. Morphological observations of trypanosomatids in different developmental stages of the life cycle show changes in the three-dimensional organization of the sub-pellicular microtubules. Little is known about the mechanisms that control this process. In *T. brucei*, the orphan kinesin TbKIN-D, which exhibits ATPase activity *in vitro*, is associated with SPMTs. RNAi targeting this protein disrupts microtubule organization, leading to the formation of additional microtubule layers and altering cell morphology. Another protein, TbKIN-C, also plays a role in organizing sub-pellicular microtubules, likely working with TbKIN-D. Studies of *T. brucei* showed that depletion of proteins such as CAP5.5/5.5V and CAP51/51V using RNAi led to the formation of multiple microtubule layers [39,40].

## Other components of the cytoskeleton

In eukaryotic cells, proteins such as actin and myosin are important components of the cytoskeleton, particularly in motility processes. It is worth noting that several genes in trypanosomatids code for these proteins. However, they exist as monomers or small oligomers that do not form typical microfilaments.

Concerning actin, genes encoding this protein have been identified in trypanosomatids, with approximately 70% homology to human actin. However, using conventional transmission electron microscopy, no actin microfilaments were seen. Additionally, no labeling is observed when using phalloidin. Genomic analysis points to the existence of components of the cytoskeleton of all trypanosomatids examined up to now. Even actin-binding proteins involved in actin regulation have been detected. Indeed, trypanosomatids possess many genes encoding actin and actin-binding proteins. Using immunolabeling, actin has been detected as patches in various regions of the protozoan, including the nucleus, kinetoplast, cortical cytoskeleton, flagellum, and flagellar pocket. They are not sensitive to cytochalasin and latrunculin and are not labeled by phalloidin [41]. In *T. cruzi*, at least four genes encode actin and several related proteins. Actin 1 is more concentrated in the anterior portion of the protozoan, especially in the region where the flagellar pocket and the cytostome are located, and that corresponds to sites where endocytic activity takes place. It was demonstrated that RNAi knockdown of the clathrin heavy chain is lethal to *T. brucei*, resulting in an enlarged flagellar pocket. Knockdown of actin in procyclic forms induces a similar phenotype [40].

The tertiary structure of actin is characterized by two main domains: the large and the small domains. Actins can assemble into protofilaments. A detailed review of the actin in trypanosomatids was conducted by [41–43]. In *T. cruzi* epimastigotes, actin is predominantly located at the base of the flagellum and as patches along the flagellum. In trypomastigotes, it is dispersed throughout the cytoplasm [41].

There is a cytoskeleton structure that encircles the flagellum at the site where it exits the cell body. In this region, flagellar pocket-associated proteins have been described, including Tb BILBO1. Knockdown of this protein results in an aberrant flagellum repositioning and a new flagellum detached from the length of the cell body. The FPC is positioned proximal and distal to a cytoskeletal structure called the hook complex (HC), which was initially described as a bilobe composed of a hook and a parallel arm [44,45]. TbCentrin2, TbCentrin4, and TbCAAP (Centrin Arm Associated Protein 1) have been localized to the arm structure. Knockdown of TbCentrin2 or TbCentrin4 prevents FAZ assembly and affects flagellum attachment. Proteins localized to the hook structure currently known are: TbSmee1, TbLRRP1, TbFPC4, and TbMORN1 (Membrane Occupation and Recognition Nexus) [44,45]. Recently, BHALIN-BILBO1 Hook Associated Linker protein (Tb927.4.3120) was described as a protein simultaneously discovered during a yeast 2-hybrid (Y2H) analysis using the full genome of *T. brucei* 927 as prey with TbBILBO1 as bait, and a proximity-dependent biotin identification using TbMORN1 as a probe [45], suggesting that BHALIN is a TbBILBO1 binding protein in very close proximity to TbMORN1, with a strategic location important for parasite viability. BHALIN 2334 3 of 24 interacts with TbBILBO1 and localizes to the part of the HC that overlaps with the FPC. The lockdown in bloodstream-form cells, dramatically distorted and lethal, is known as the “BigEye” phenotype. In addition, treatment of these cells with cytochalasin B, an inhibitor of actin polymerization, reduces endocytic activity [46,47].

Trypanosomatids are considered highly divergent eukaryotes that still conserve an actomyosin system [46–49]. Genes coding for two classes of myosins were identified in trypanosomatids. One myosin, designated as TbMyo1, is localized between the nucleus and the kinetoplast. Its silencing by RNAi leads to enlargement of the flagellar large cytosolic compartment, alterations in clathrin localization, and inhibition of endocytic activity. Tb Myo 1 has a large cytosolic pool and the ability to translocate actin filaments *in vitro*. Using several approaches, it was clearly shown that an actomyosin system, with myosin TbMyo1 as the active molecular motor, is localized near the endosomal system and can associate with it in the bloodstream form of *T. brucei*. It was also shown that both actin and Myo1 play a role in the organization and integrity of the endosomal system: RNAi-mediated depletion of Myo1 and treatment with latrunculin A disrupted endomembrane integrity. Another myosin, Myo 21, was identified in *Leishmania* and localized to the proximal region of the flagellum, where the basal body is located [46]. Myo21 interacts with actin, and a decrease in its expression results in modification of the length of the flagellum and loss of the PFR. Other myosins identified include MyoA, MyoB, MyoC, MyoD, MyoE, MyoF, and MyoG, some of which are associated with the cytostome–cytopharinx complex [22,50,51].

Light and electron microscopy, together with biochemical and biophysical assays, were used to explore the relationship between the actomyosin and endomembrane systems. The class I myosin (TbMyo1) had a large cytosolic pool, and its ability to translocate actin filaments *in vitro* was shown here for the first time. TbMyo1 exhibited a strong association with the endosomal system and was additionally found on glycosomes. At the endosomal membranes, TbMyo1 colocalised with markers for early and late endosomes (TbRab5A and TbRab7, respectively), but not with the marker associated with recycling endosomes (TbRab11). Actin and myosin were simultaneously visualized for the first time in trypanosomes using an anti-actin chromobody. Disruption of the actomyosin system using the actin-depolymerizing drug latrunculin A resulted in a delocalization of both the actin chromobody signal and an endosomal marker, and was accompanied by a specific loss of endosomal structure. This suggests that the actomyosin system is required for maintaining endosomal integrity in *T. brucei* [47]. Myosins are motor proteins that comprise a large, diverse family and are important for a broad range of functions. Two myosin classes, I and XIII, were previously assigned in Trypanosomatids, based mainly on studies of *T. cruzi*, *T. brucei*, and Leishmania major, and of important human pathogenic species; seven orphan myosins were identified in *T*. *cruzi* [47,48]. Studies by de Souza and co-workers [49] show that the great variety of *T. cruzi* myosins is also present in some closely related species and in *Bodo saltans*, a member of an early-diverging branch of Kinetoplastida. Therefore, these myosins should no longer be considered “orphans.” They proposed the classification of a kinetoplastid-specific myosin group into a new class, XXXVI. Moreover, phylogenetic data suggest that a large repertoire of myosin genes was present in the last common ancestor of trypanosomatids and *B. saltans*, mainly due to multiple gene duplications. These genes have since been predominantly maintained in synteny in some species, and secondary losses explain the current distribution. They also found two interesting genes that were clearly derived from myosin genes, demonstrating that potentially redundant or useless genes, rather than simply being lost, can serve as raw material for the evolution of new genes and functions.

Recently, several proteins have been identified in the epimastigotes of *T. cruzi* [51]. They used a combination of CRISPR–Cas-9-mediated endogenous tagging, fluorescently labeled overexpression constructs, and endocytic assays to identify the first cytostome–cytopharynx protein, designated CP1. Using immunoprecipitation and mass spectrometry, two other proteins were identified as associated with CP1 and designated CP2 and CP3.

## The cytoskeleton of apicomplexa

Apicomplexa protists comprise a large group of eukaryotic microorganisms that cause diseases of high medical relevance in humans and animals. It is important to highlight the genera *Plasmodium*,* Toxoplasma*,* Cryptosporidium*, and *Eimeria.* They exhibit a complex evolutionary cycle in which the different stages display an exuberant cytoskeleton with distinct features.

**Figure 5:**
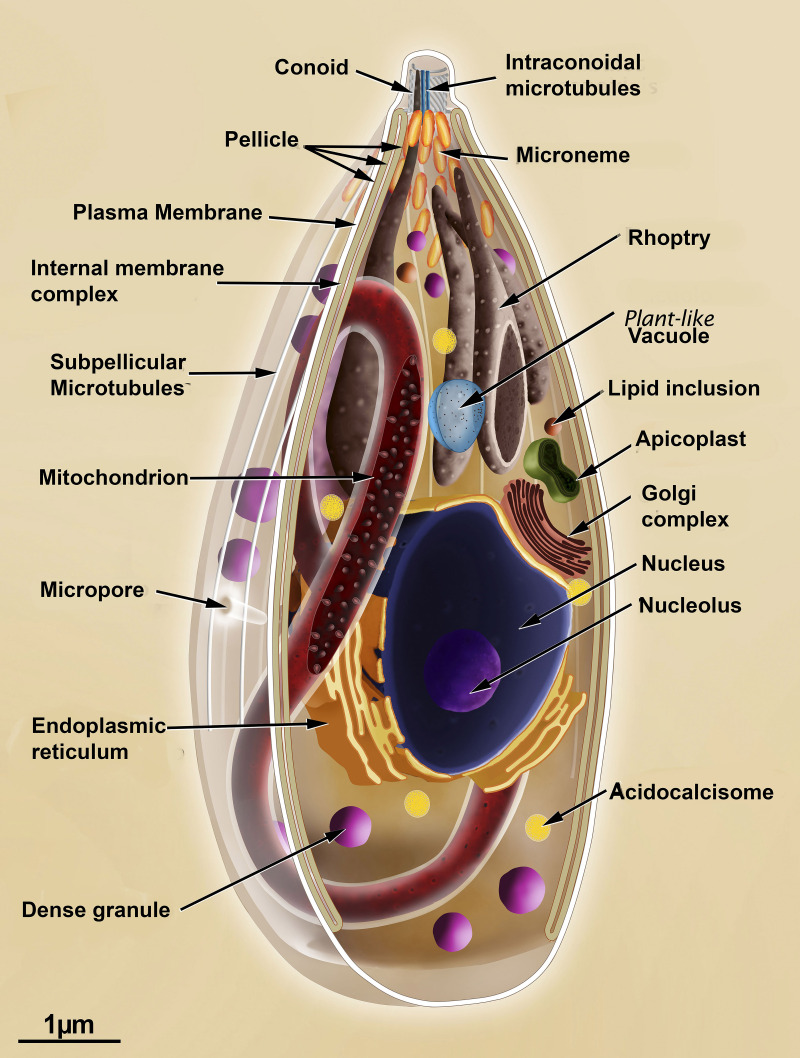
Schematic view of a longitudinal section of a tachyzoite of *Toxoplasma gondii.* The most important structures and organelles are indicated. This scheme was developed by some of the authors [55].

A key characteristic of the infective stages is the presence of a membrane complex comprising a plasma membrane and a complex of internal membranes, with two juxtaposed membrane units, situated immediately below the plasma membrane and spanning most of the protist’s body, except for the most anterior and posterior extremities. In the apical portion, the presence of secretory organelles, such as micronemes and rhoptries, and the conoid, is notable [52–56]. Figure 5 illustrates a general scheme of the organization of a tachyzoite of *T. gondii* [55].

## Microtubule system

**Figure 6:**
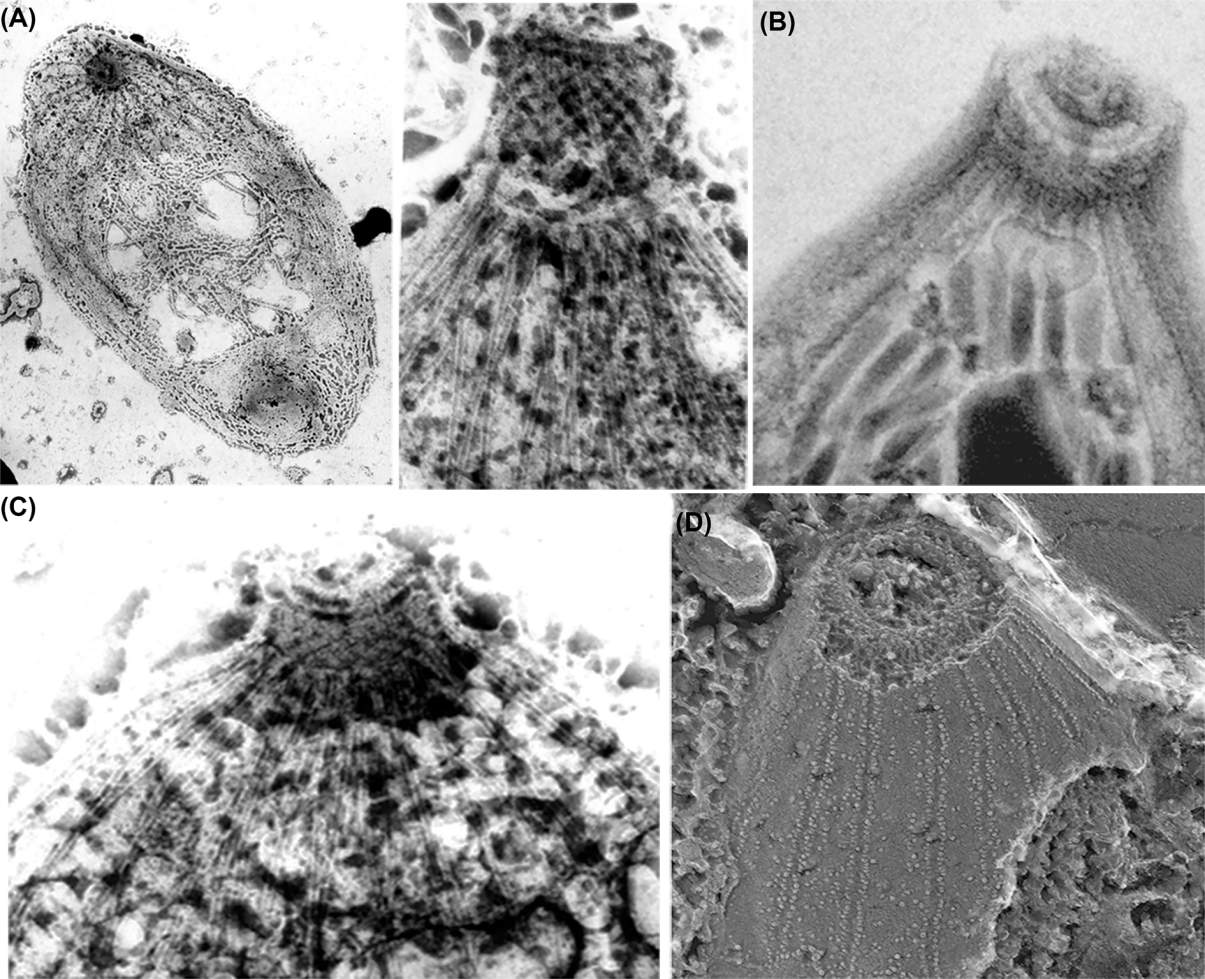
The conoid of *Toxoplasma* tachizoites The conoid of Toxoplasma tachizoites. a,c. Negative staining of whole tachyzoites. The images show the conoid and the sub-pellicular microtubules originating from the polar anel and radiating towards the posterior region. The organization of the conoid is also illustrated [52,53]. (b), tangential thin section of the conoid showing the emergence of the sub-pellicular microtubule as well as the complex organization of the conoid. (d) freeze-fracture and deep-etching replica of the anterior region, revealing the linear organization of intramembranous particles on the fracture faces of the inner membrane complex and the network of filaments (actin) in the inner portion of the conoid (unpublishedd image obtained by Lengruber, Vommaro, and De Souza). All images are from one of the authors (WS)

We identify at least four components: the sub-pellicular microtubules, located just below the inner membrane complex (IMC); those that make up the conoid; those of the nuclear spindle; and those of the flagellum present in microgametes. The system of sub-pellicular microtubules is certainly striking in Apicomplexa (Figures 5 and 6). The number of these microtubules varies among species: 22 in *T. gondii*, 2 in *Plasmodium* merozoites, and 11 in sporozoites of the same species. These microtubules exhibit a unique organization, with transverse striations visible in negative-staining contrast preparations [52–54,56–60]. They are extremely resistant to temperature variations and even to compounds that prevent polymerization. It is believed that this resistance is due to their association with proteins located in the tubular lumen [61]. They originate from a polar ring at the anterior end of the protozoan, corresponding to a zone of microtubular organization that is characterized by the important participation of γ-tubulin, as clearly shown by expansion microscopy [59]. These microtubules are composed of protofilaments, the number of which varies [60]. Several MAPs have been described, including some that are located in the microtubule lumen [61]. Components of the IMC include Glideosome-associated proteins with multiple membrane spans, known as GAPMs [61]. Morphological analysis using conventional freeze-fracture and deep-etching showed that the fracture faces of the IMC, a complex structure composed of interconnected plates, facilitated by the participation of specific proteins, revealed the presence of a linear array of intramembranous particles longitudinally oriented to the major axis of the protozoan in a disposition similar to that of the sub-pellicular microtubules (Figure 6).

## Conoid

**Figure 7:**
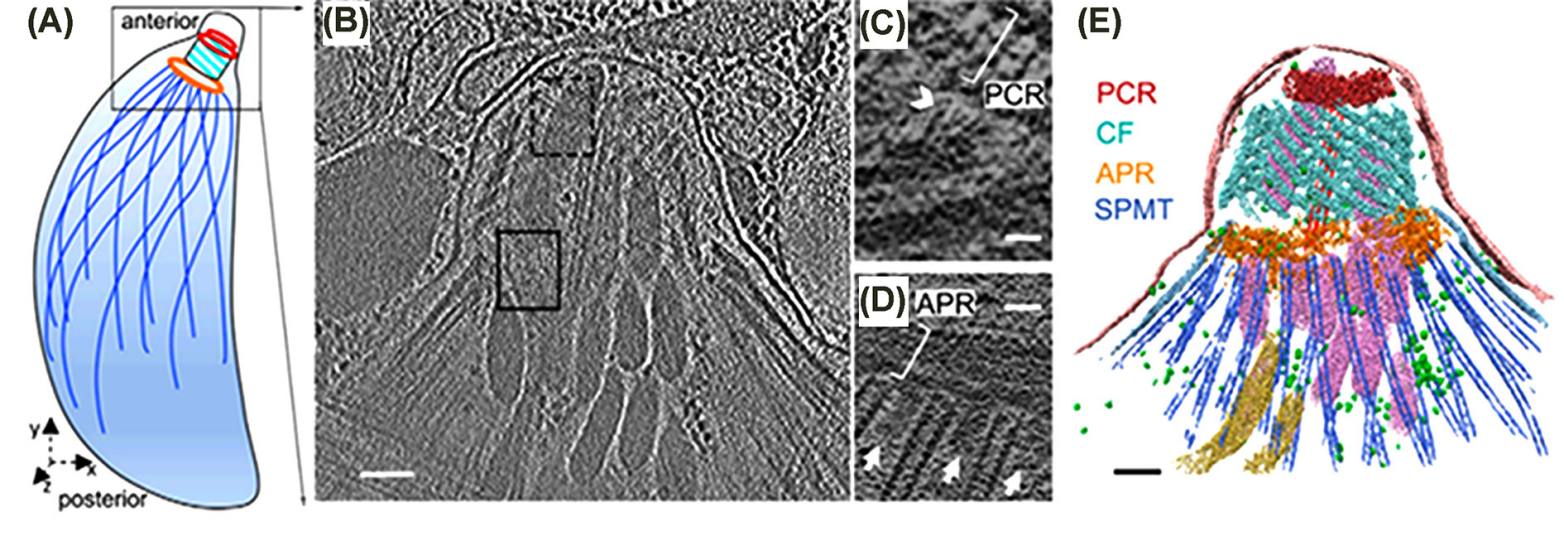
Three-dimensional organization of the apical complex in *Toxoplasma* tachyzoites reveals the SPMT and conoid architecture (**A**) Cartoon of the *Toxoplasma* tachyzoite, the life stage of which has been imaged by cryo-ET in B and annotated in E. (**B**) Tomographic slice of a representative apical complex of an intact tachyzoite recorded with VPP (Vortex Phase Plates) optics in an electron microscope. The dotted and solid rectangles indicate the regions of the apical complex, represented in greater detail in (C) and (D), using different z-slices (scale bar: 100 nm). (**C**) A zoomed-in view of a tomographic slice in the dashed black rectangle of B, showing two CFs in the *xy* plane, their anterior ends extending toward a PCR (white bracket) with filamentous density (a white arrowhead) between them (scale bar: 25 nm). (**D**) Portion of a tomographic slice in the black rectangle of B, showing the SPMTs in the *xy* plane, and their association with the APR (white bracket). Note columnar densities (white arrows) emerging from the APR and positioned between the SPMTs (scale bar: 25 nm). (**E**) Three-dimensional segmentation of the tomogram shown in (B), including the PCR (red), CFs (cyan), APRs (golden), SPMTs (blue), intraconoidal microtubules (red), micronemes (pink), rhoptries (yellow), and plasma membrane (pale pink) (scale bar: 100 nm). (After [71]). Image with permission as published as Open Access from PNAS, distributed under Creative Commons Attribution-NonCommercial-NoDerivatives License 4.0 (CC BY-NC-ND)

**Figure 8:**
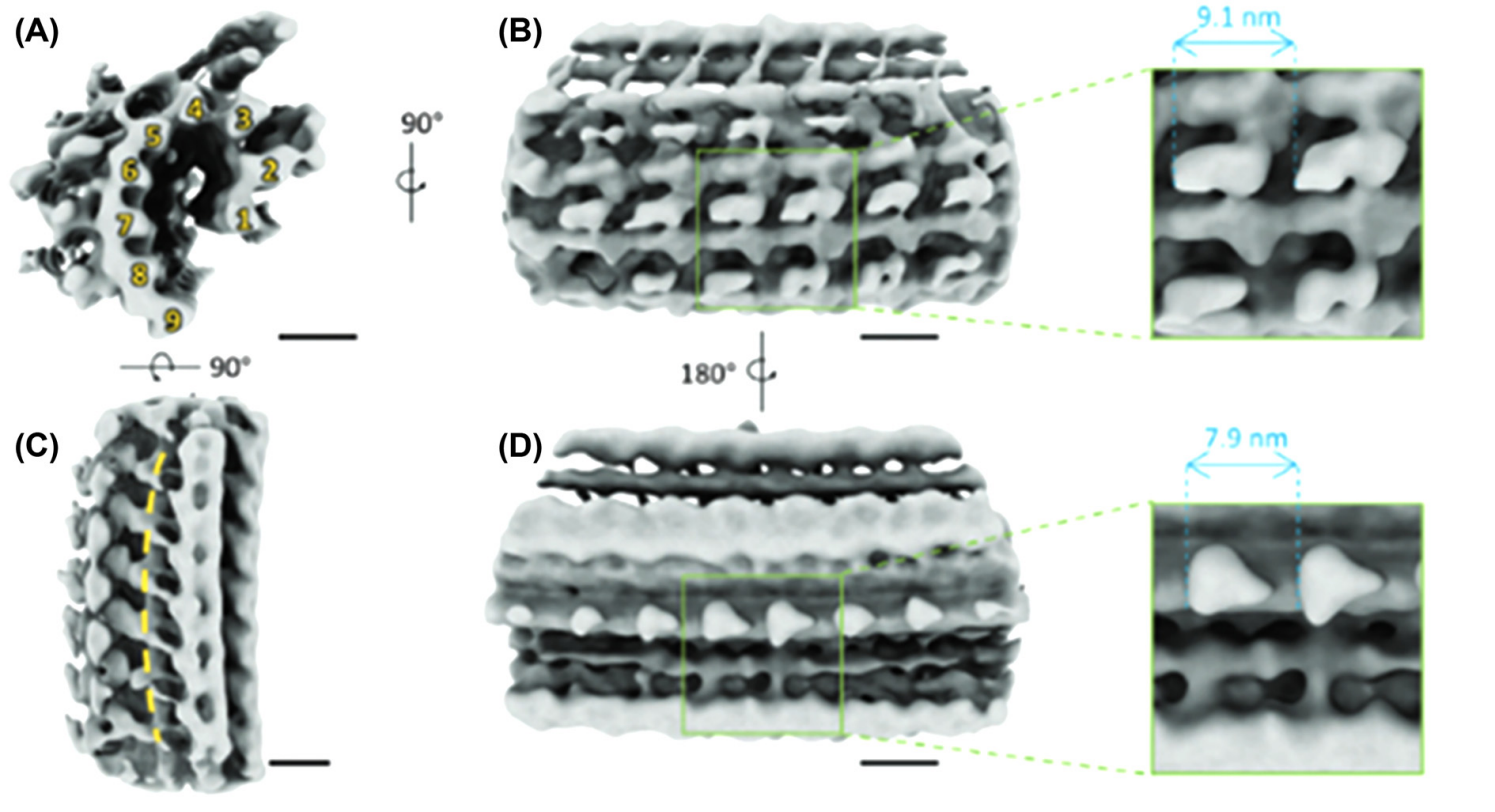
Structural analysis of the *T. gondii* conoid fiber in the retracted state (**A**) Cross‐section of the conoid fiber segment. Nine tubulin protofilaments are arranged into a comma shape, as indicated by the yellow numbers. (**B–D**) Density map of the conoid fiber segment from different views as indicated. A representative repeated patch is marked with a green box, and the patch size is measured from both the (B) convex side and the (D) concave side. The yellow dashed line along the segment indicates the curved structure (C); scale bars: 10 nm. After [72]. Image with permission as published as Open Access from Adv. Science.

The conoid complex, as currently defined, comprises the conoid, intraconoidal microtubules (ICMTs), preconoidal rings (PCRs), and the apical polar ring (APR) [56]. The barrel-shaped conoid is striking in some species of Apicomplexa, such as *T. gondii*, where it appears as a complex structure composed of annular rings that form the polar rings (Figures 5-8). It represents a complex organization of specialized, relatively short microtubules, in which the tubular structure is barely apparent and consists of only 9 protofilaments. It consists of about 15 microtubules, also called fibrils, in a spiral arrangement [52,53,56,60,62–64] as well as two intraconoidal microtubules. Recent studies using cryomicroscopy have revealed new details. For example, the negative side of the microtubule faces the most apical region of the conoid. The conoid is a dynamic structure that appears either retracted or extruded during parasite invasion of a cell. This extrusion involves the actin-myosin complex [65–69]. Several proteins have been reported to be associated with the conoid [69]. Little is known about the formation of a new conoid during the initial phases of endodyogeny in *T. gondii*.

A crucial component of the conoid is the conoidal ring, composed of proteins and often exhibiting periodicity. In the case of the apical collar, there is a clear association with the internal membrane complex that, as mentioned before, does not reach the most anterior portion of the protozoan. The other polar rings also act as actin nucleation sites [69–74]. The number of these rings varies among species: 3 in Toxoplasma and Neospora, and 2 in *Cryptosporidium* [64,69]. Several proteins that interact with conoid components have been identified, including protein 6, CGP, and myosin H [63–65], which may play a role in initiating parasite motility. Some of these proteins have been identified in species previously considered to lack a conoid, such as *Plasmodium* [69], suggesting the presence of conoid-related structures in other members of the Apicomplexa.

Transmission electron microscopy images reveal a denser material associated with the cytoplasmic portion of the IMC, forming a fibrillar network that remains poorly characterized morphologically. Proteins called alveolins are associated with this structure. The IMC-1 protein is an alveolin, and its knockout results in significant changes in the protozoan’s shape. Other proteins are also associated with this structure, establishing some association with the SPMTs [72,74–79].

Several proteins have been identified as linking microtubules to the IMC [61,62,65,67,74–79]. Additionally, there are indications of an association with other cellular structures. One of these proteins is PhiL1-GAPM. Its deletion leads to changes in the protist's morphology and to reduced infectivity [64,75]. Another relevant protein is SPM3, which connects the microtubules to the IMC. GAP45, which connects the IMC complex to the glideosome complex, is also relevant [75].

**Figure 9:**
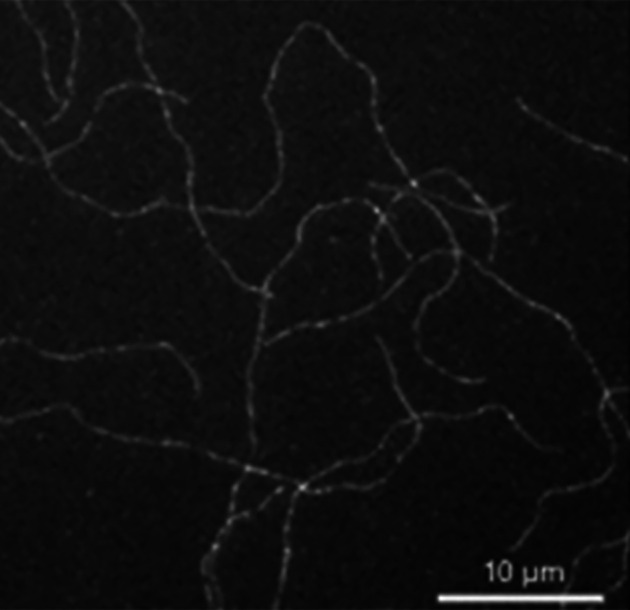
*Toxoplasma* actin1 Imaging of *Toxoplasma* actin1 polymerized *in vitro* (after [79]). Permission as open access from Nature Communications, licensed under a Creative Commons Attribution 4.0 International License.

The motility of Apicomplexa protists is facilitated by gliding motility, a type of movement that involves association with a substrate. An important component is actin, which is nucleated by interaction with formins, leading to the formation of small oligomers that assemble into short microfilaments, which are difficult to visualize by traditional electron microscopy. However, recent studies using (a) filament-binding chromobodies [76,79] and (b) cells treated with jasplakinolide, which is an actin filament stabilizer [76], show filaments in the space between the plasma membrane and the IMC. It is essential to note the presence and participation of myosin, which is anchored to the IMC through several proteins, including four GAPs, facilitating the movement-generating interaction with actin and enabling speeds of approximately 10 micrometers per second (Figure 9).

## The cytoskeleton of *trichomonads*

*Trichomonas vaginalis* and *Tritrichomonas foetus* are sexually transmitted parasites responsible for urogenital infections. They provoke abortions and other health problems in humans (*T. vaginalis*) and cattle (*T. foetus*). Both cause trichomoniasis, a widespread sexually transmitted infection; in humans, it is the most common non-viral infection globally. *T. foetus* leads to abortions and health issues in livestock, resulting in significant veterinary and farm losses. Alongside typical eukaryotic organelles, such as the nucleus, Golgi apparatus, endoplasmic reticulum, and lysosomes, these parasites lack mitochondria; instead, they possess hydrogenosomes that generate ATP and molecular hydrogen. Notably, their cytoskeleton is remarkably complex.

## Microtubular structures

**Figure 10:**
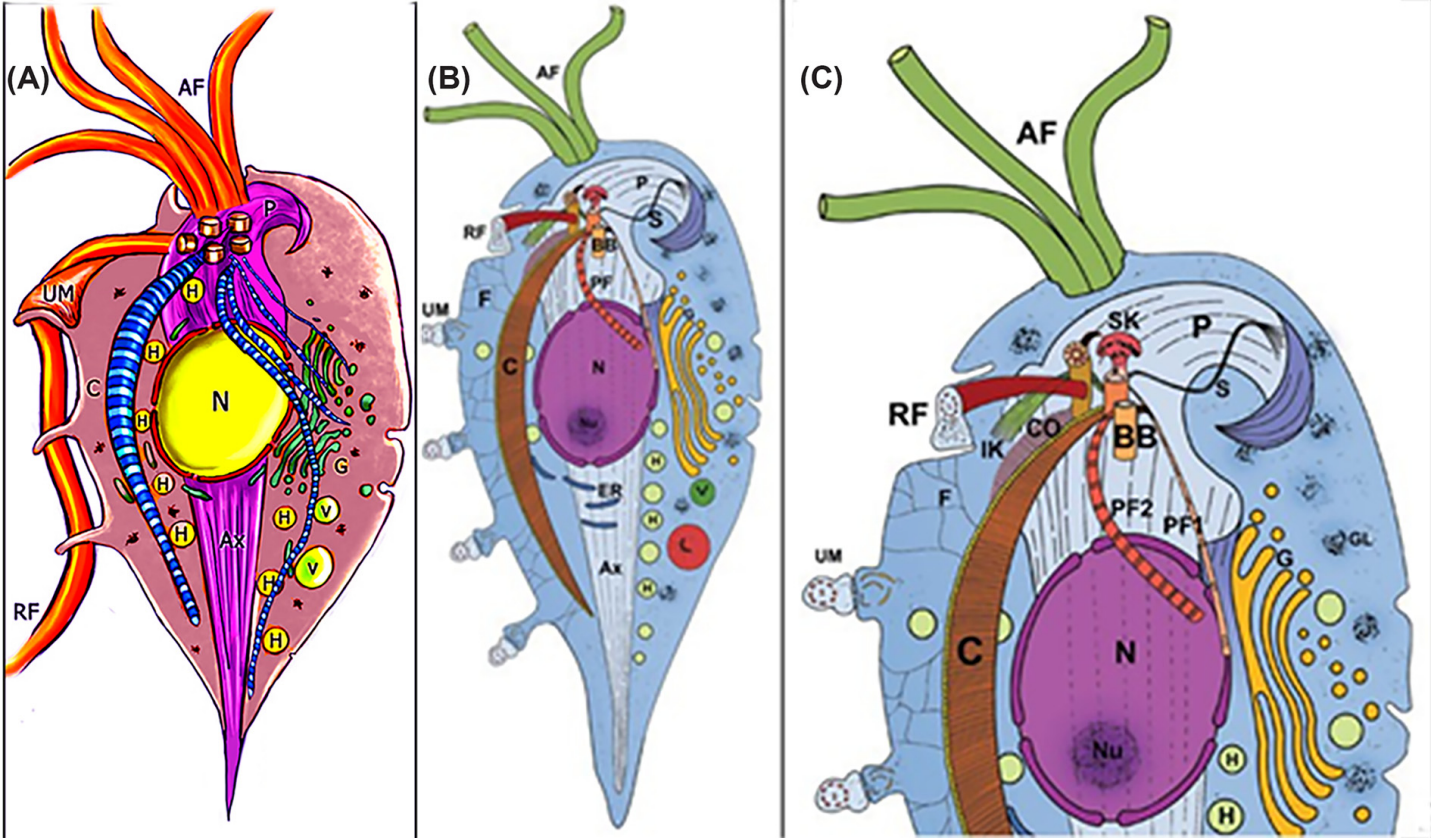
*Trichomonas* schemes *T. vaginalis* (**A**) and *T. foetus* (**B** and **C**) schemes. AF, anterior flagella; Ax axostyle; BB basal body; C, costa; Co comb; ER endoplasmic reticulum; F filaments connecting the costa to the plasma membrane; G Golgi; GL glycogen granules; H, hydrogenosome; K infra-kinetosomal filament; L lysosome; N nucleus; Nu nucleolus; P, pelta; PF parabasal filament; *PF_1_* parabasal filament number 1; RF, recurrent flagellum; S sigmoid filaments; SK supra-kinetosomal body; UM undulating membrane; V vacuole (All schemes are from one of the authors (MB). (A) Adapted from [80]; (**B** and **C**) From [81].

**Figure 11:**
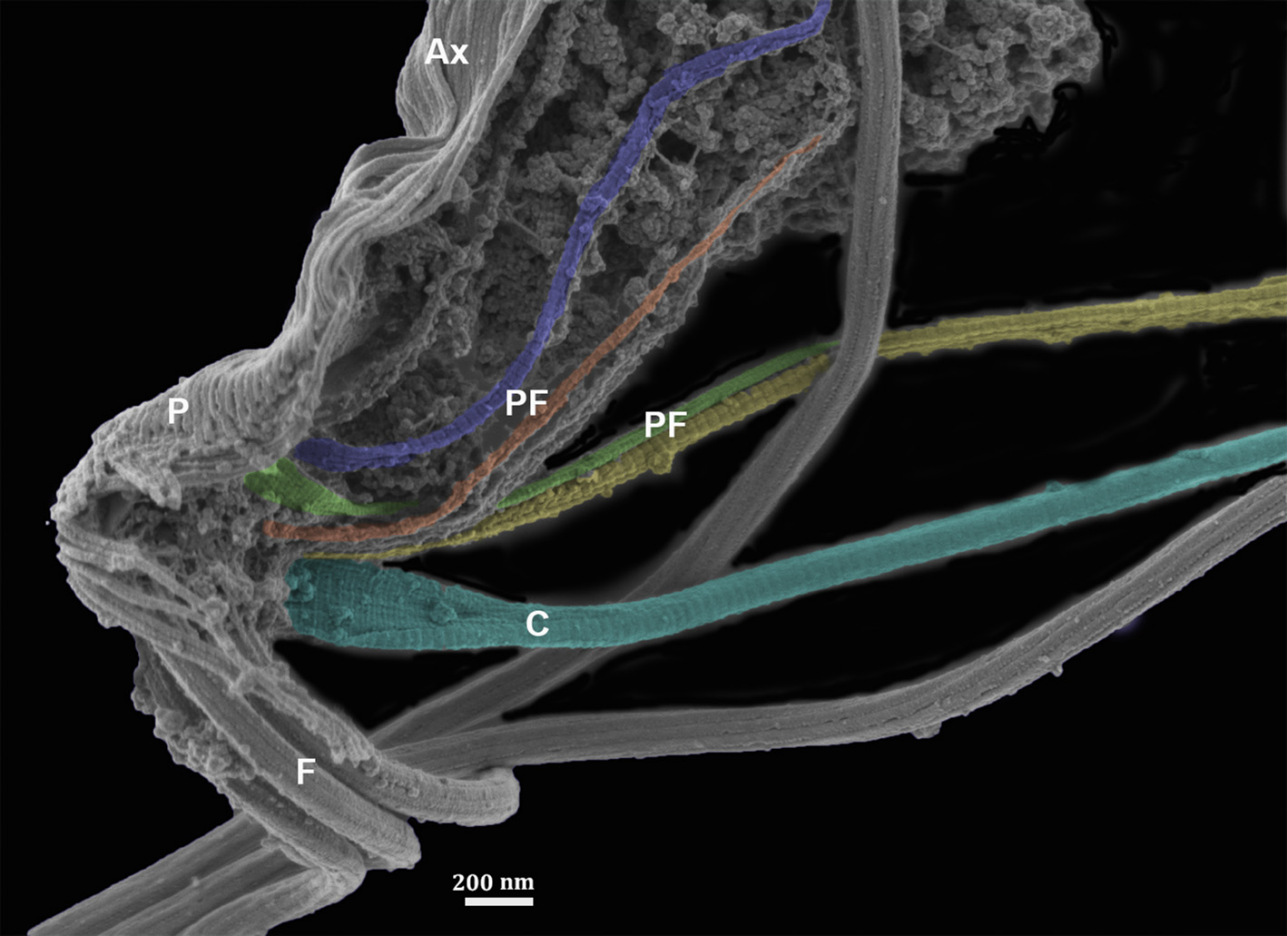
Ultra-high-resolution SEM of the isolated cytoskeleton of *T. vaginalis.* The four striated fibers are colored yellow (PF1), orange (PF2) (PF1), green (PF3), and purple (PF4). Axostyle (Ax). Flagella (F), Pelta (P). (Benchimol, unpublished).

**Figure 12:**
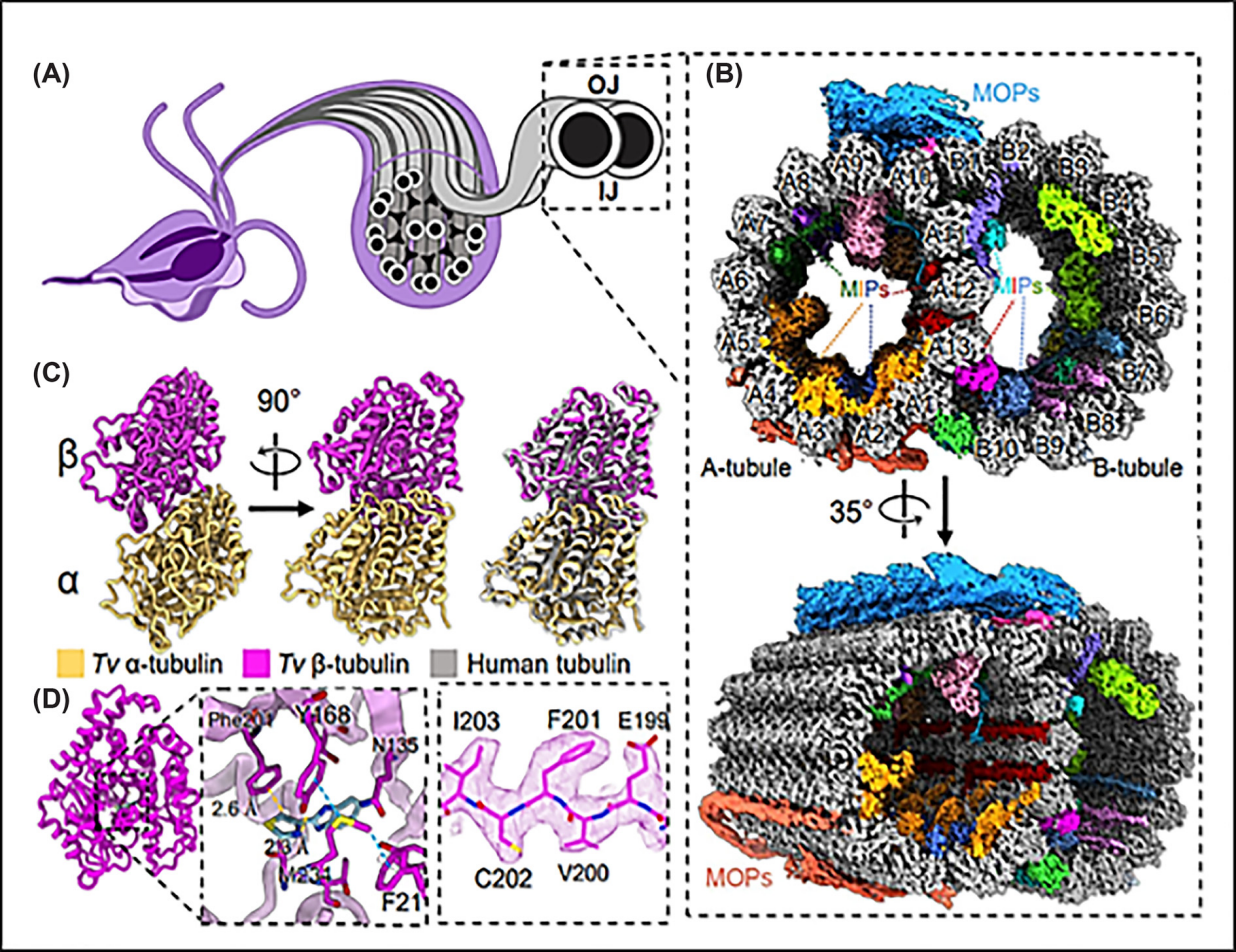
Cryo-EM reconstruction of the doublet microtubules from T. vaginalis. (**A**) Cryo-EM reconstruction of the DMTs from *T. vaginalis*. Axoneme and (**B**) cross-section with microtubule inner (MIPs) and outer (MOPs) proteins (several colors). (**C**) Atomic models of α- and β-tubulin, superimposed with human tubulin (right). (**D**) Alternate view of β-tubulin (left) and docked thiabendazole molecule (blue) fit into the putative binding site with adjacent residues shown (right) with cryo-EM map density. IJ: inner junction; OJ: outer junction. (From [85]). Permission granted as Open Access from Nature Communications. This article is licensed under a Creative Commons Attribution 4.0 International License.

**Figure 13:**
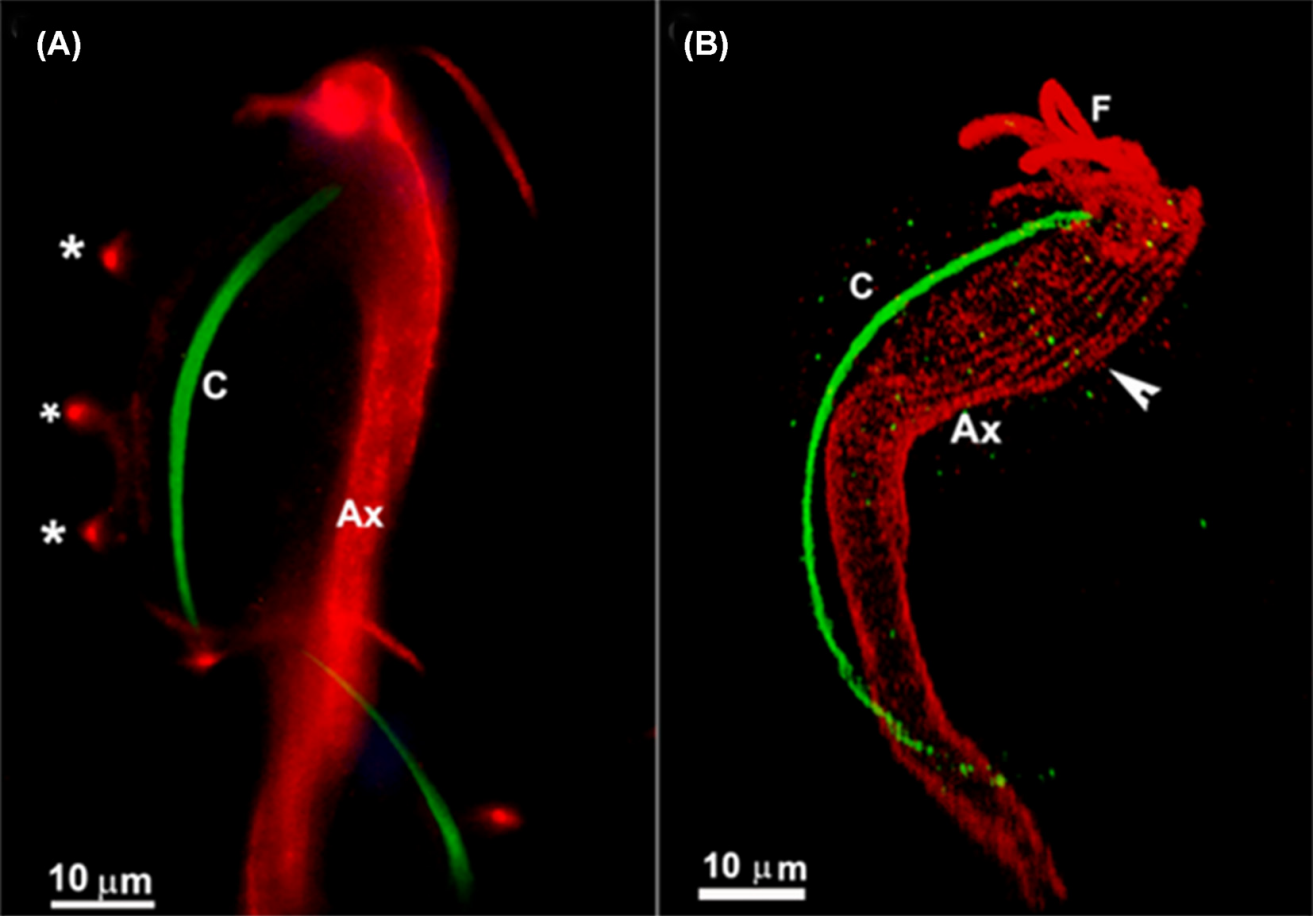
Fluorescent microscopy of *T. foetus* after the expansion Fluorescent microscopy of *T. foetus* after the expansion (**A** and **B**). The axostyle (Ax) is labeled red by anti-tubulin, while the costa (C) is identified in green with the polyclonal anti-costain1 antibody. DAPI labels the nucleus (N). F, flagella; asterisks, undulating membrane; arrowhead, microtubules. (After [93]). Images are from one of the authors (WS).

**Figure 14:**
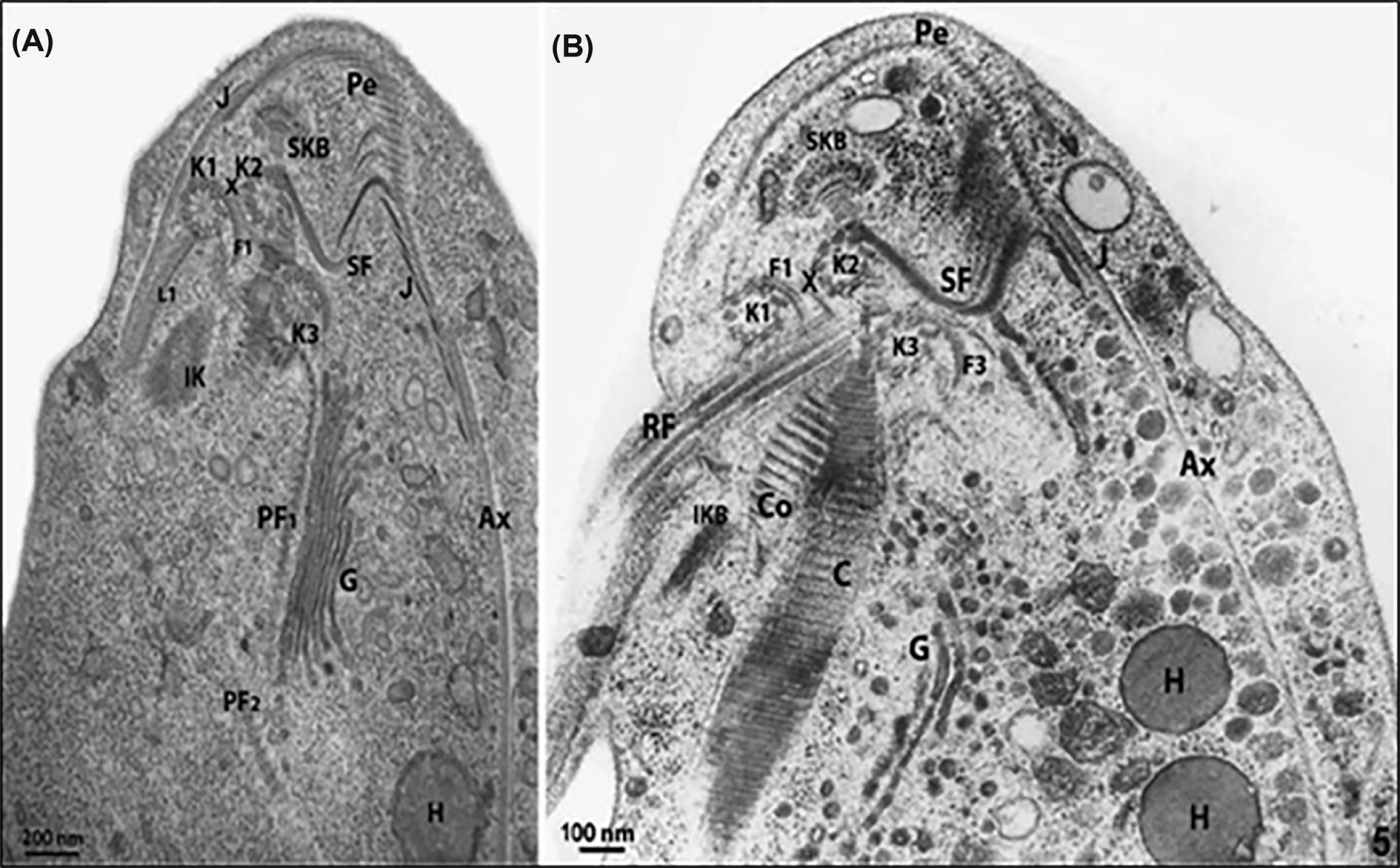
Anterior region of* T. foetus* (**A** and **B**) Routine thin sections of *T. foetus* through the anterior region. These two sequential sections display the complex structures that form the cytoskeleton. The basal bodies (kinetosomes) K1, K2, and K3 are seen. Originating from kinetosome 1 (K_1_), lamella 1 (L_1_) is located toward the posterior region. The sigmoidal filaments (SF) emerge from kinetosome 2 toward the pelta (Pe). Comb (Co). Pelta (Pe); Axostyle (Ax); Pelta-axostylar junction (J). Recurrent flagellum (RF); Sigmoid filaments (SF). The supra-kinetosomal) body (SKB) appears as a stalk connected to the basal body 2. Infra-kinetosomal body (IKB). The parabasal filaments (PF_1_–PF_2_) follow the Golgi complex (G). H, hydrogenosome. (a, b). Benchimol, unpublished.

**Figure 15:**
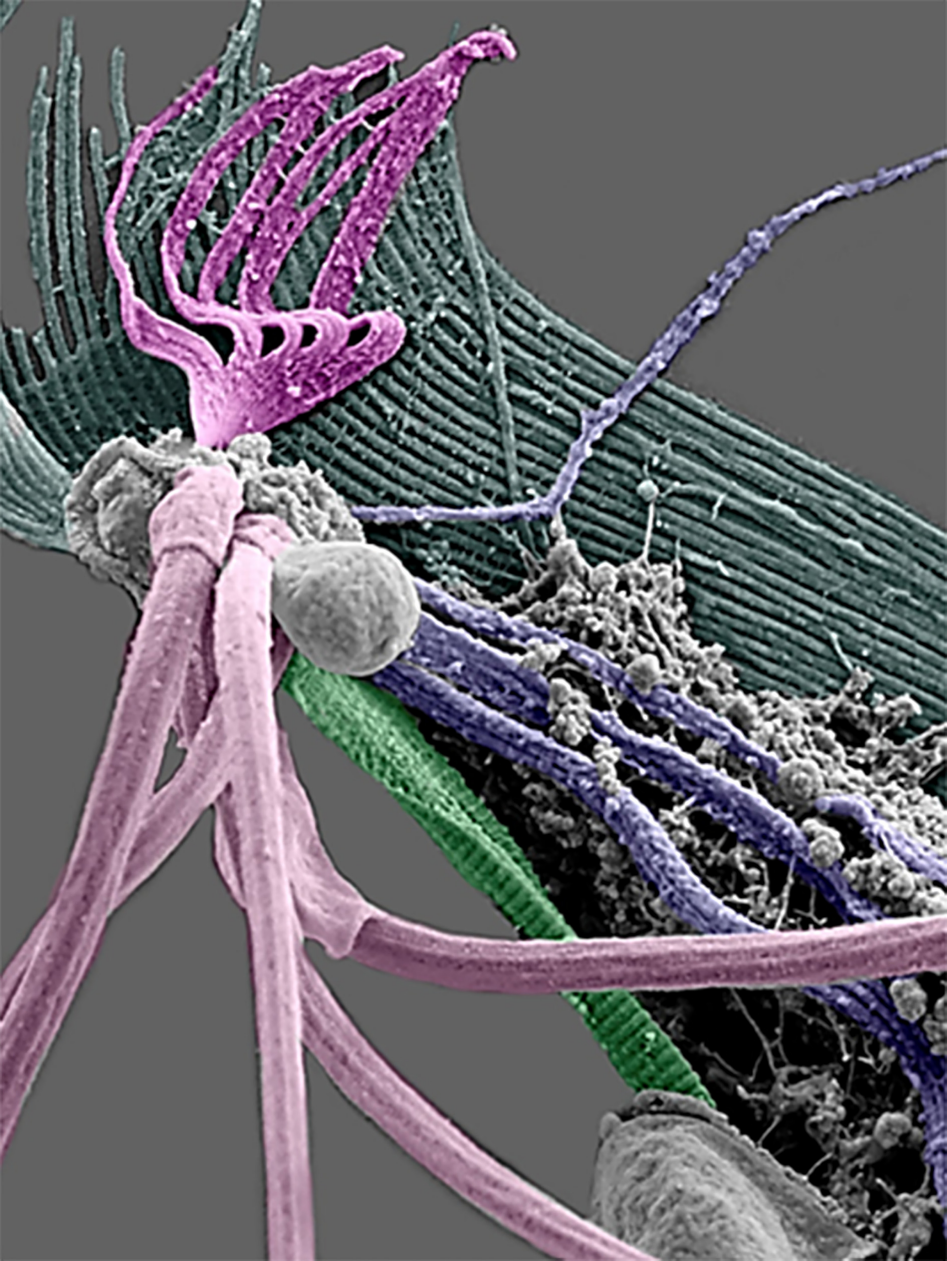
High-resolution SEM of the isolated *Trichomonas vaginalis* cytoskeleton shows a fan-out of a sigmoidal fiber (S) Ax, axostyle; C, costa; F, flagella; PF, parabasal filaments. Benchimol, unpublished.

**Figure 16:**
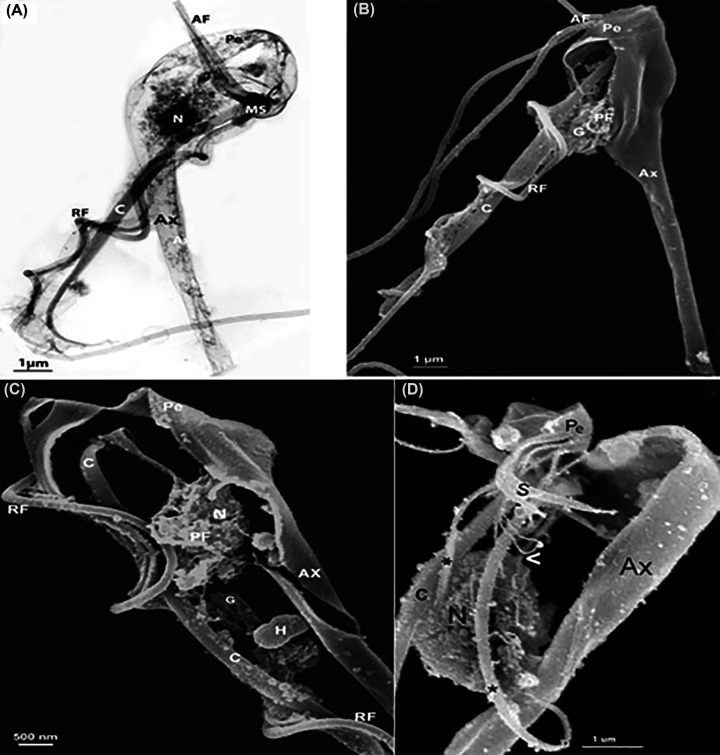
Internal view of *T. foetus* (**A**) Internal view of *T. foetus* using high-voltage transmission electron microscopy (HVTEM) or detergent treatment to remove the plasma membrane and exhibit the cytoskeleton (**B–D**, the F2). Anterior flagella (AF), recurrent flagellum (RF); axostyle (Ax); Costa (C); Nucleus (N); Pelta (Pe); S, sigmoid; PF, parabasal filament (Benchimol unpublished).

The primary components of the cytoskeleton include the pelta-axostyle system, which is composed of stable microtubules and other proteinaceous structures (Figures 10-16). *T. vaginalis* has five flagella: four anterior and one, the recurrent flagellum, forming the undulating membrane. *T. foetus* has three anterior and one recurrent flagellum, which are internalized under stress, forming pseudocysts without true cell walls [80]. Both species feature several little-known structures originating from their basal bodies. They consist of fibrous proteins with unknown functions. Their composition is difficult to analyze because isolating individual fibers is challenging, as other fibers are often included during cellular fractionation. Among these, we can mention the sigmoid filament, the X structure, several appendages, and rootlets that emerge from the basal bodies (Figures 10 and 11).

## Basal body (kinetosomes)

The axonemes of the flagella present characteristics typical of eukaryotic flagella, with a 9 + 2 arrangement of microtubules, and they originate from basal bodies located in the most anterior region of the cell. Unlike *T. vaginalis*, *T. foetus* exhibits the supra-kinetosomal and infra-kinetosomal bodies, as well as the comb (Figure 10).

Current studies are focused on characterizing the ultrastructural and biochemical properties of *Trichomonas* cytoskeletal components. The use of ultrafast freezing and high-resolution electron microscopes, combined with tomography, has led to a better comprehension of these structures [81].

The axostyle, composed of longitudinal microtubules running from the anterior to the posterior region of the cell, narrows to a thin, membrane-covered tip. In *T. foetus*, it contains about 150 microtubules, each 24 nm in diameter and spaced 40 nm apart, connected by filamentous bridges with a uniform 25 nm spacing [82]. The pelta supports the wall where flagella emerge [82] (Figure 11). Functionally, the axostyle supports the cell axis and participates in karyokinesis, making it crucial to the biology of trichomonads [83,84].

Centrin, also called caltractin, is a 20-kDa acidic protein present in centrosomes and basal bodies of flagellated or ciliated cells, including mammals. As a member of the EF-hand calcium-binding family, it shows calcium-sensitive contractility. In unicellular organisms, centrin associates with specific fibrous structures but is absent from the costa, parabasal fibers, and pelta-axostyle complex. The *T. vaginalis* genome encodes centrin genes.

Recently, one group [85] analyzed the proteins in the flagellar microtubules of *T. vaginalis* using cryomicroscopy. The authors identified 29 unique proteins, of which 18 are present in the internal microtubules and 9 in the external microtubules of the axonemes (Figure 12).

## Filamentous structures

Structures such as parabasal filaments and the costa exhibit periodicity, forming a protein framework that supports the recurrent flagellum and creates the undulating membrane. The costa, parabasal filaments, sigmoid, and rootlet appendages originate from the region of the basal bodies [81,82].

## Costa

**Figure 17:**
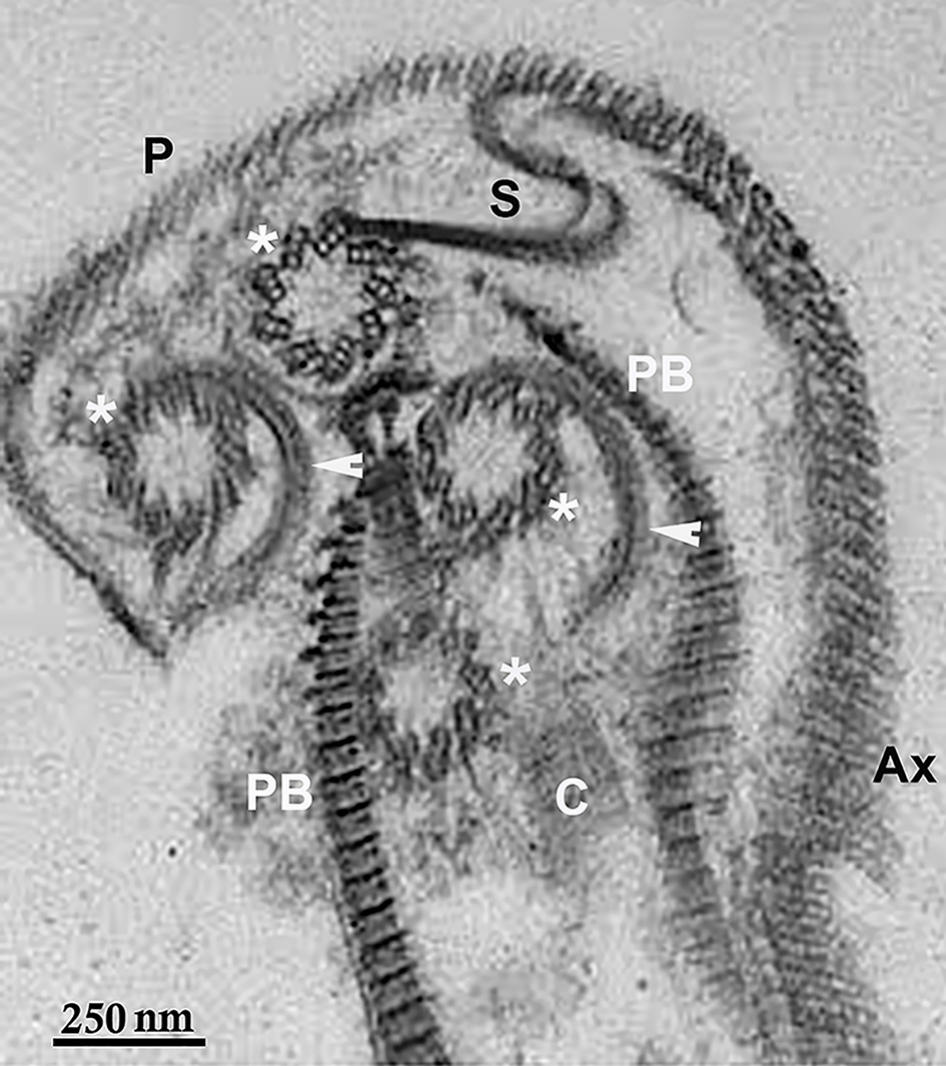
TEM of isolated cytoskeleton from *T. vaginalis* The plasma membrane was removed with detergent, exhibiting the basal bodies (asterisks), the parabasal filaments (PB), the costa (C), microtubules of pelta (P), the axostyle (Ax), and the sigmoidal filament. Arrowheads point to rootlets that emerge from the basal bodies. Benchimol, unpublished.

*Trichomonas* have striated root fibrils, including the costa and four parabasal filaments, which display periodic thin, wide bands of complex filament arrays [83,86]; (Figures 13-17). The non-contractile costa provides mechanical support to the undulating membrane [82,83]. There are two costa types by striation, size, and shape: type A (in *T. foetus*) and type B (in *T. vaginalis*, Trichomonas, Pentatrichomonas), with type B showing a herringbone pattern and 42–60 nm periodicity. This periodicity may vary depending on the fixation method.

Atractophores, or microtubule-organizing centers (MTOCs), are found at the base of the costa under kinetosome #2 and form the spindle [86]. used a monoclonal antibody to identify P477, an atractophore-associated protein in *T. vaginalis*. According to the TIGR genome database, P477 is a large coiled-coil protein that likely serves as a structural scaffold, without catalytic or binding activity, facilitating transport to the Golgi centrosomal region. Similar proteins are present in other protists.

Research by Viscogliosi and Brugerolle, as well as studies from our team, utilized monoclonal antibody production [87–89] and biochemical methods, such as cell fractionation to isolate highly purified costa fractions. These analyses demonstrated that B-type costa proteins in trichomonads primarily comprise polypeptides with molecular weights between 100 and 135 kDa, values comparable to those of A-type costa proteins. SDS–PAGE revealed multiple protein bands in the costa, with major bands at apparent molecular weights of 122, 115, 112, 93, 87, 82, 59, 44, 41, 32, and 26 kDa. These methodological approaches enabled the effective separation of primary protein constituents, specifically those ranging from 100 to 150 kDa [87]. Using monoclonal antibodies, five polypeptides—measuring 135, 125, 114, 88, and 47 kDa—were identified via immunoblotting, with immunofluorescence microscopy confirming their localization to the costa [87]. Notably, no immunological cross-reactivity with other trichomonad genera was detected, indicating unique biochemical profiles consistent with structural distinctions. Nonetheless, partial protein similarity suggests that certain proteins share epitopes [87].

Previous studies using proteomic analysis of enriched costa fractions have identified 44 hypothetical proteins lacking conserved domains [88–90]. More recently, a study identified and characterized the first protein in the costa of *T. foetus*, called costain-1 [91]. The group identified two additional proteins in the *T. foetus* costa: costain-2 (11810) and costain-3 (32137). They observed that each protein had a slightly different localization in the costa striation pattern [91]. Additionally, these proteins were predominantly α-helical and likely formed a coiled-coil structure. The authors of this study suggested a structural role based on domains identified by software (including myosin, Centrosomal Protein 2, and the chromosome segregation protein SMC). They used AlphaFold3 models as templates to model these proteins. Expansion microscopy was employed to accurately locate costain-1 within the costa of *T. foetus* [92].

## Actin

A substantial amount of actin, its isoforms, and nine separate actin genes have been detected in *T. vaginalis* [93,94]. Actin filaments are observed in the cortical region of trichomonads, particularly during ameboid morphological states and when interacting with host cells [94–96]. Alpha-actinin is an F-actin cross-linking protein found in multiple organisms. Research indicates the presence of an atypical α-actinin protein in *T. vaginalis*, characterized by a unique amino acid sequence within its rod domain. Immunofluorescence assays show that it co-localizes with actin at the cell periphery in pseudopods of adhering cells [95–97]. One group [94] reported the distribution of actin, filamin, and myosin throughout the cytoplasm, with filamin more concentrated at the cell periphery.

## Clockwise filaments

Several filaments and lamellae originate from the kinetosome region (Figures 10, 14, 15 and 17). Each kinetosome forms clockwise-oriented filaments: Kinetosome #1 generates filament F1 and the marginal lamellae (ML) of the UM from its left, while filament IK1 emerges to the right of the ML. The X filament connects kinetosome #2 and F1 (Figure 14), and kinetosome #3 initiates the periodic filament F3 (Figure 14).

The supra-kinetosomal bodies (SKB) (Figures 9 and 13) are stalked structures unique to the Tritrichomonadinae. These bodies are associated with basal body #2 at the region of sigmoid filament attachment (Figures 13-15). Although the precise function and molecular composition of the SKB remain undetermined, they do not react with anti-tubulin antibodies.

## Sigmoidal filaments

The sigmoidal filament (SF) (Figures 14-17), also known as the F2 filament, is composed of parallel filaments arranged in a curved pattern that links kinetosome #2 to the pelta, spreading at the initial bend. Initially, it forms a trunk, which then divides into seven to ten sheets [81]. SF tracks alongside the axostyle microtubules for some distance, ending near, though not directly at, the pelta-axostyle junction within the cytoplasm. It has been observed to be centrin-positive and actin-negative, with its function currently unknown [81]. The sigmoidal filament measures about 1 μm wide and 900 nm long.

Tomographic imaging was performed by two groups: [98] and [81]. Findings from one group [81] indicate that sigmoid filaments consist of multiple individual filaments. According to another group [98], the curvature of each sigmoid filament gradually decreases.

## The parabasal filaments (PBFs)

*T. vaginalis* displays four PBFs: PF1, PF2, PF3, and PF4 (Figures 16 and 17) [81]. All PFs originate in the kinetosome region, near the base of the costa. PF1 begins between kinetosomes #2 and #3, while PF2 shares an origin with the base of the costa and arises from kinetosomes #2 and R (from the recurrent flagellum). Recent studies using tomography and high-resolution scanning electron microscopy described the relationship between the PBFs and the Golgi complex [81]. PBFs exhibit a periodic structure featuring alternating electron-dense and electron-lucent regions, where dense areas contain four thin lines. In trichomonads, PFs are situated above the nucleus and below the Golgi complex [81].

Earlier literature used the term “parabasal apparatus” to describe this association with the Golgi complex. Its function is posited to support this organelle, though current evidence does not conclusively confirm this hypothesis.

## Proteomic and genome sequence

De Jesus group [99] worked in a application of two-dimensional electrophoresis and matrix-assisted laser desorption/ionization and Time-of-flight mass spectrometry for proteomic analysis of *T. vaginalis*. The parasite genome comprises six chromosomes and approximately 60,000 protein-coding genes [100]. Although the genomes of *T. vaginalis* and *T. foetus* have been examined [100,101], studying their cytoskeletal proteomics remains difficult due to the complexity of the trichomonad cytoskeleton, which complicates isolation of pure components—a challenge addressed in studies on costa proteins [89–92].

The draft genome sequence of the sexually transmitted pathogen *T. vaginalis* identified genes for several flagellar proteins [100]. These include PF16 homolog, PF20 homolog, LF4, IFT20 homolog, IFT57 homolog, IFT172 homolog, RIB43, and RIB72. Genes encoding motor proteins, such as dynein and kinesin, were identified, including the Dynein light, intermediate, heavy, and β chains. Tubulins identified in the genome included α, β, γ, Δ, and ϵ forms. The γ-tubulin complex proteins comprised GCP-2 and Spc97/Spc98 homologs. Additional proteins detected included Microtubule Decoration proteins (CLASP homologs, MAP1 light chain, and MAP-65 homolog), Microtubule Capping proteins (EB family and CLIP-170), and Microtubule-Severing proteins (Katanin p60 and p80 subunits).

Previous work [102] found that parabasalian parasites possess cytoskeletal proteins with intermediate filament characteristics. Proteomic analysis of *Tetratrichomonas gallinarum* revealed 203 proteins similar to those in *T. vaginalis*, many featuring coiled-coil regions. Expressing a single *T. vaginalis* protein in *T. gallinarum* produced striated filaments. The study indicates that while these protist filament-forming proteins share structural features with metazoan intermediate filaments, they likely evolved independently or from distinct evolutionary paths, highlighting the uniqueness of eukaryotic cytoskeletal filaments.

One group [103] described an unusual F-actin capping protein that may regulate cytoskeletal functions critical for *T. vaginalis* colonization, suggesting a new actin-mediated pathway in parasite morphogenesis, attachment, and motility.

*Trichomonas* relies on actin and tubulin proteins, encoded by 10 actin and 11 tubulin genes, for cell shape and division [104]. investigated the expression and characterization of these genes, revealing actin throughout parasite structures and tubulin in areas such as the axostyle, flagella, and mitotic spindle. Tubulin-positive nanotubular structures emerged during late cytokinesis, highlighting the essential and diverse roles of the actin and tubulin gene families in intercellular interactions and cell division.

## The cytoskeleton of *Giardia*

The genus *Giardia* includes several species that infect a wide range of hosts. Among these, *Giardia intestinalis* stands out as the species that infects mammals, including humans and domestic animals such as dogs and cats, causing giardiasis. This disease is characterized by symptoms such as vomiting, nausea, and, most notably, diarrhea. In children and immunocompromised individuals, the infection can result in severe malnutrition, which may lead to both physical and cognitive impairments. *Giardia* is one of the most common waterborne pathogens, with the highest incidence occurring in areas with inadequate sanitation and poor water treatment systems.

**Figure 18:**
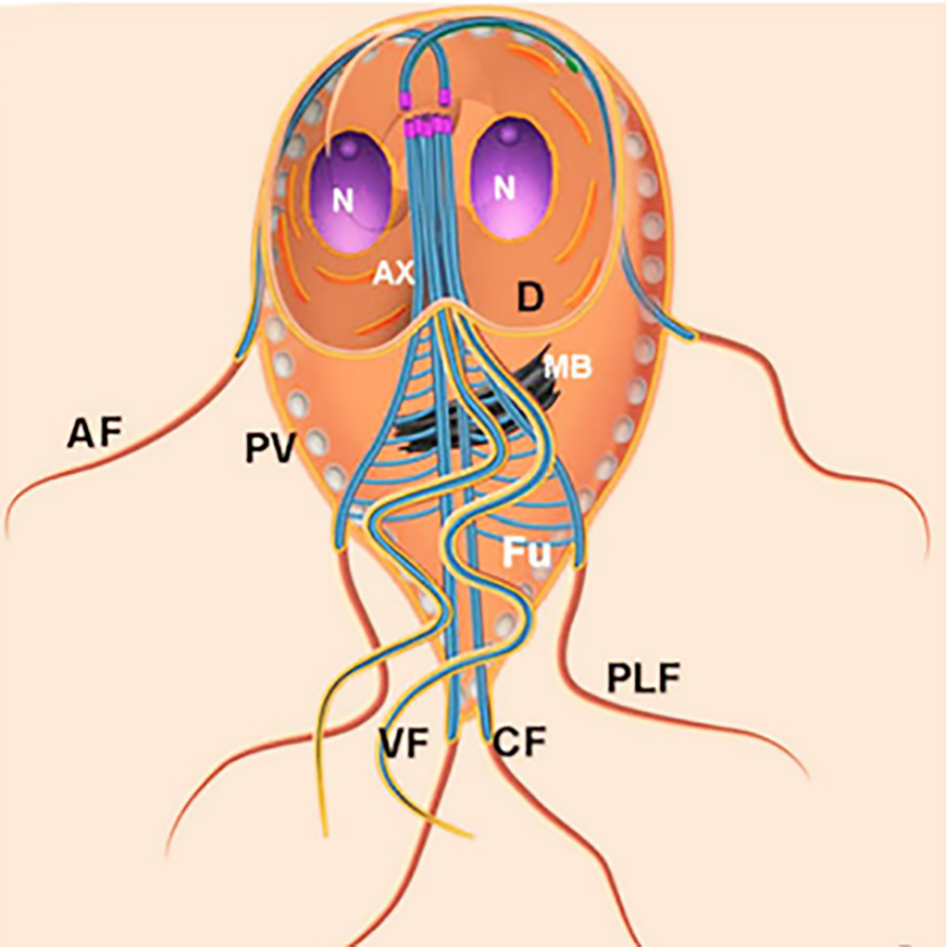
Schematic view of a longitudinal section of a trophozoite of *G. intestinalis* The most important structures and organelles are indicated. This scheme was developed by one of the authors (MB). AF, anterior flagella; VF, ventral flagella; CF, caudal flagella; PLF, posterior-lateral flagella; N, nucleus; Ax, axonemes; MB, median body; Fu, funis; PV, peripheral vesicle; D, ventral disc. Image from the authors.

**Figure 19:**
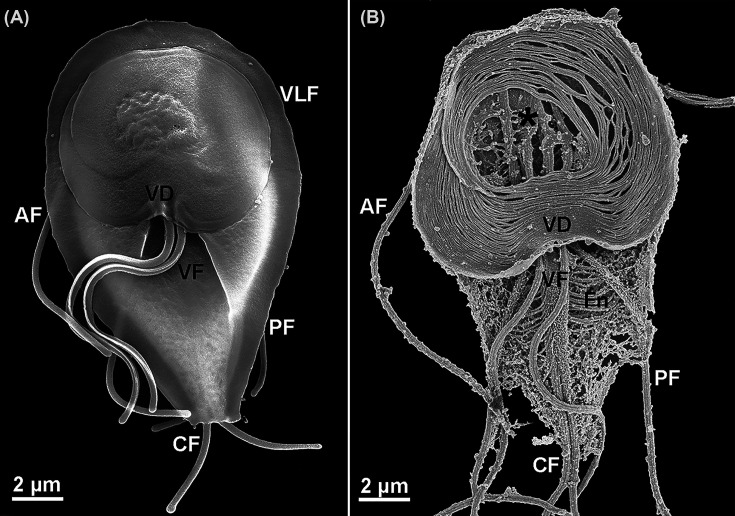
High resolution scanning electron microscopy of a trophozoite of *G. intestinalis.* (**A**) Trophozoite was prepared using a conventional fixation method. The ventral disc (VD), ventro-lateral flange (VLF), and flagella are visible. (**B**) Trophozoite following membrane extraction with 2% NP-40 detergent, revealing internal cytoskeletal structures including the ventral disc (VD), axonemes, basal bodies (*), and the funis (Fn). AF, anterior flagellum; PF, lateral-posterior flagellum; CF, caudal flagellum; VF, ventral flagellum. Images from the authors [108]. Open Access (OA) agreement.

Among the structures present in this protozoan are two active nuclei, endoplasmic reticulum, ribosomes, and glycogen granules (Figure 18). A classical Golgi apparatus has not been identified. Additionally, specialized organelles, such as peripheral vesicles, are involved in endocytic processes [105,106]. *Giardia* lacks typical mitochondria but instead contains mitosomes, which are considered highly reduced mitochondrial remnants [107]. The cytoskeleton structures include a ventral disc, a median body, four pairs of flagella, and funis, which will be described in detail below [108] (Figures 18 and 19A and B). Since protozoa motility, attachment, and division are critical steps in establishing infection, and are mediated by the cytoskeleton, understanding the organization and precise regulation of these structural elements is essential for elucidating the mechanisms underlying the parasite’s pathogenicity.

## Giardia cytoskeleton structures

### Ventral disc

**Figure 20:**
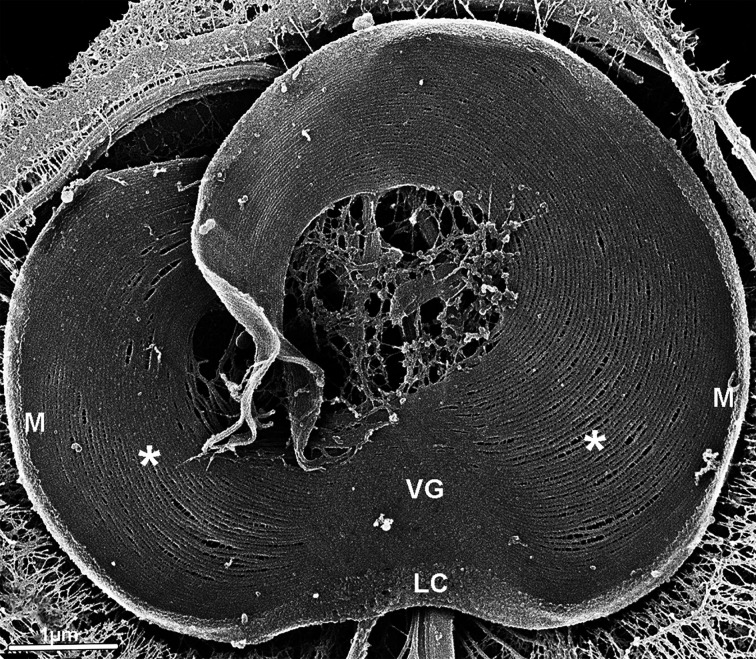
Helium ion microscopy image of the cytoskeleton of the *G. intestinalis* ventral disc Microtubule–microribbon complexes appear more tightly packed in the ventral groove (VG) and margin (M) regions compared to the more widely spaced central region (*). Note the globular aspect of the lateral crest (LC). ((Images from the authors. Image of the authors [113]. Elsevier Permission for reuse.

Scanning electron microscopy of the ventral surface of *G. intestinalis* trophozoites reveals the ventral disc, a concave structure localized in the anterior region of the cell (Figures 18 and 19). Its spiral shape is also discernible, resulting from the ordered arrangement of its components. The ventral disc is composed of spaced microtubules, associated with microribbons—trilaminar structures that connect perpendicularly to the microtubules and are linked to one another by cross-bridges [109–111]. At the center of the spiral defined by the microtubule-microribbon complex lies a region devoid of these cytoskeletal elements, named the bare area. In this region, a protrusion containing cytoplasmic components such as glycogen granules, ribosomes, and vesicles can be observed [109]. The ventral disc is externally surrounded by a fibrous lateral crest [112,113] (Figure 20).

Advances in bioimaging technologies, such as electron tomography and cryotomography, have allowed the observation of structural heterogeneity in the ventral disc, indicating regional variations in the organization of its elements. At the beginning of the spiral, in the region known as the dorsal overlap zone, and at the disc margin, the microtubules display a more compact arrangement in contrast to other areas of the disc (ventral overlap zone and disc body) [114]. Morphological differences were also observed in the microribbons: in the dorsal overlap zone and margin, they are 30–40 nm long, whereas in other regions they are greater than 50 nm [114]. Subsequent analysis of cells with extracted plasma membranes using ion microscopy combined with High-resolution scanning electron microscopy revealed additional structural details [111,112]. This work showed that the cross-bridges at the disc margin and in the ventral canal region are smaller and more resistant to rupture. In both zones, the spacing between the microtubule–microribbon complexes is considerably reduced compared to other regions [111]. Taken together, these findings support the conclusion that the ventral disc is a highly specialized and heterogeneous structure composed of distinct domains and possibly functional roles (Figure 20).

**Figure 21:**
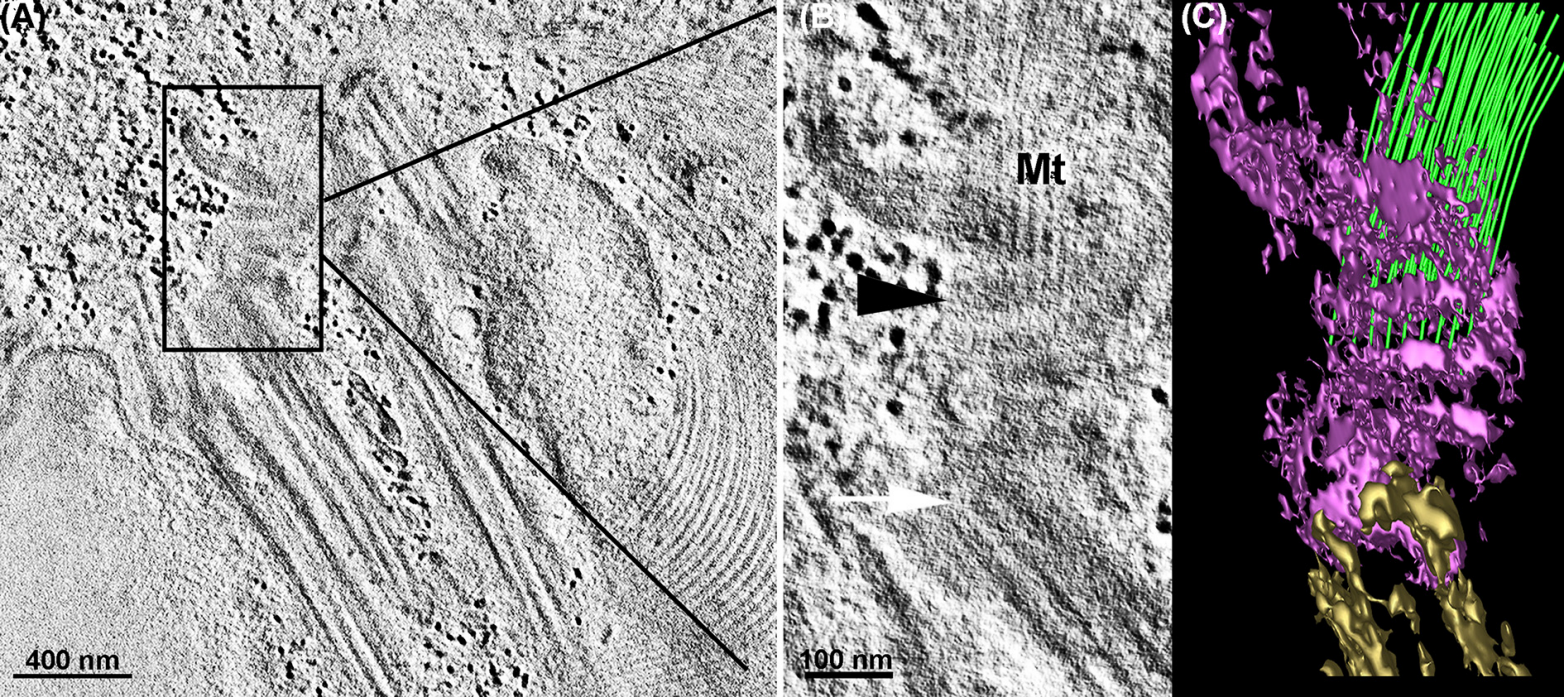
Basal body region of *Giardia intestinalis* visualized by TEM tomography (**A** and **B**) Virtual slices from the tomogram showing basal bodies (white arrow), the banded collar (black arrowhead), and microtubules (Mt). (B) a higher magnification of the area highlighted in (**A** and **C**) Corresponding 3D reconstruction illustrating the spatial relationship between these components. Brown: basal body; purple: banded collar; green: microtubules. Images from the authors [108]. Open Access (OA) agreement.

In addition to its structural components, the origin of the ventral disc’s microtubules is notable. Observations from negative-stain samples indicate that these microtubules nucleate from three electron-dense bands localized at the bare area, known as the banded collar [115]. This region was later defined as the “microtubule nucleation zone” [114]. High-resolution scanning microscopy and electron tomography analyses have shown that the banded collar is composed of three to four interconnected segments, arranged in a spiral configuration, and is closely associated with the basal bodies of the caudal and the ventral flagella of the left tetrad (as observed from a dorsal view of the cell) [108,113]. At the center of this spiral, ventral disc microtubules emerge from multiple segments, supporting the hypothesis that the banded collar acts as a microtubule-organizing center for this specialized cytoskeleton structure [108] (Figure 21). This suggestion is further corroborated by immunocytochemical analysis that revealed positive staining for γ-tubulin at the site of microtubule emergence from the banded collar [108,113]. Notably, ultrastructural analyses using high-resolution microscopy techniques have also indicated that microtubules nucleated at the banded collars connect with others formed near the perinuclear region [110]. These findings reinforce the notion that the nucleation of disc microtubules can occur in a distributed manner, involving multiple organizing centers, which may contribute to the structural and functional complexity of the ventral disc.

Tubulins are the primary components of the *G. intestinalis* cytoskeleton, existing in various isoforms and undergoing diverse post-translational modifications. In the ventral disc, acetylated α-tubulin is particularly abundant, a modification commonly associated with increased microtubule stability [108,113]. In addition, polyglycylated tubulin has also been identified in the disc structure, suggesting further evidence for cytoskeletal regulation and specialization [116,117].

Electron tomography analyses have revealed protein complexes associated with the ventral disc microtubules, including microtubule inner proteins (MIPs) and MAPs, indicating a specialized molecular organization [110]. Proteomic analyses performed from isolated ventral discs, as well as immunofluorescence-based approaches using specific antibodies, have enabled the identification of a set of disc-associated proteins. Among these are α-giardins, homologous to annexins; β-giardin, Δ-giardin, and SALP-1, all related to SF-assemblies [118–120]. Additionally, the γ-giardin and median body protein (MBP) were also identified [120,121]. Most disc-associated proteins lack homology to proteins from other organisms, making it challenging to predict their functions. Novel structural components of the ventral disc and lateral crest in *Giardia intestinalis* have been published [122]. However, the subcellular localization of some of these proteins has been confirmed by fluorescence microscopy using GFP-tagged proteins, allowing visualization within specific disc components [123,124]. Functional approaches based on CRISPR-mediated genetic interference were used to investigate the biological roles of several newly identified DAPs [124], providing new insights into the organization and dynamics of the ventral disc. For example, knockdown of DAP5188 or DAP6571 results in defects in the disc's hyperstability, whereas depletion of DAP7268 leads to aberrant disc morphology [125].

Significant progress has also been made regarding the mechanism of attachment mediated by this structure. Previously, two main hypotheses were proposed. The first one stated that attachment was driven by a suction force generated by the beating of the ventral flagellum [126,127]. However, advances in molecular approaches and high-resolution bioimaging technologies have challenged this model. Notably, assays using cells lacking flagellar motility have shown that attachment is independent of flagellar beating [128,129]. The motility of the ventral flagellum may play a role during the initial stages of adhesion, facilitating substrate sensing and positioning [129].

Another function of the ventral disc involves cell division. According to [130], the disc may actively participate in karyokinesis by exerting compressive forces on the nuclear envelope, thereby assisting nuclear segregation and advancing mitotic progression in the parasite.

## Flagella and associated structures (funis)

One of the peculiar features of *G. intestinalis* is the presence of eight flagella. These are organized into four pairs, arranged in two tetrads, and are named according to their region of externalization on the cell surface. Accordingly, they are classified as anterior, posterolateral, ventral, and caudal flagella. In addition to their positioning, the flagella also differ in length and function. The ventral flagella have a unique characteristic that distinguishes them from the others: they bear a thin membrane projection, resembling a fin, filled with fibrillar material known as the paraflagellar or paraxial rod [109,123].

As observed in other eukaryotic models, the basic structure of the flagella of the *G. intestinalis* follows the typical arrangement of nine peripheral microtubule doublets surrounding a central pair. Proteomic analysis of the parasite’s isolated flagella, performed by LC-MS, identified key structural components, including tubulins, dynein heavy and light chains, nexin links, and radial spoke proteins [131]. Additional cytoskeletal elements, such as ankyrin-repeat and coiled-coil domain-containing proteins, were also identified. The flagellar protein profile also included enzymes from both the glycolytic and arginine deiminase pathways, as well as proteins associated with actin dynamics [131]. Notably, the flagellar proteome was enriched for membrane transporters, vesicle-trafficking proteins, and signaling molecules, including tyrosine kinases, calcium transporters, and mitogen-activated protein kinases. This molecular profile suggested that the *G. intestinalis* flagellum may also function as a sensory organelle [131]. Importantly, several α-giardins—proteins related to human annexins that display calcium-dependent binding to phospholipids—were identified in the flagella [131,132]. In addition, post-translational modifications of tubulin, such as acetylation, polyglycylation, monoglycylation, and glutamylation, were detected, reflecting the complex regulation of flagellar microtubule dynamics [116,117,131]. Regarding associated proteins, one group [133] reported the *in vivo* function of a kinesin-2 homologue in G. intestinalis and determined its structure by high-resolution crystallography.

**Figure 22:**
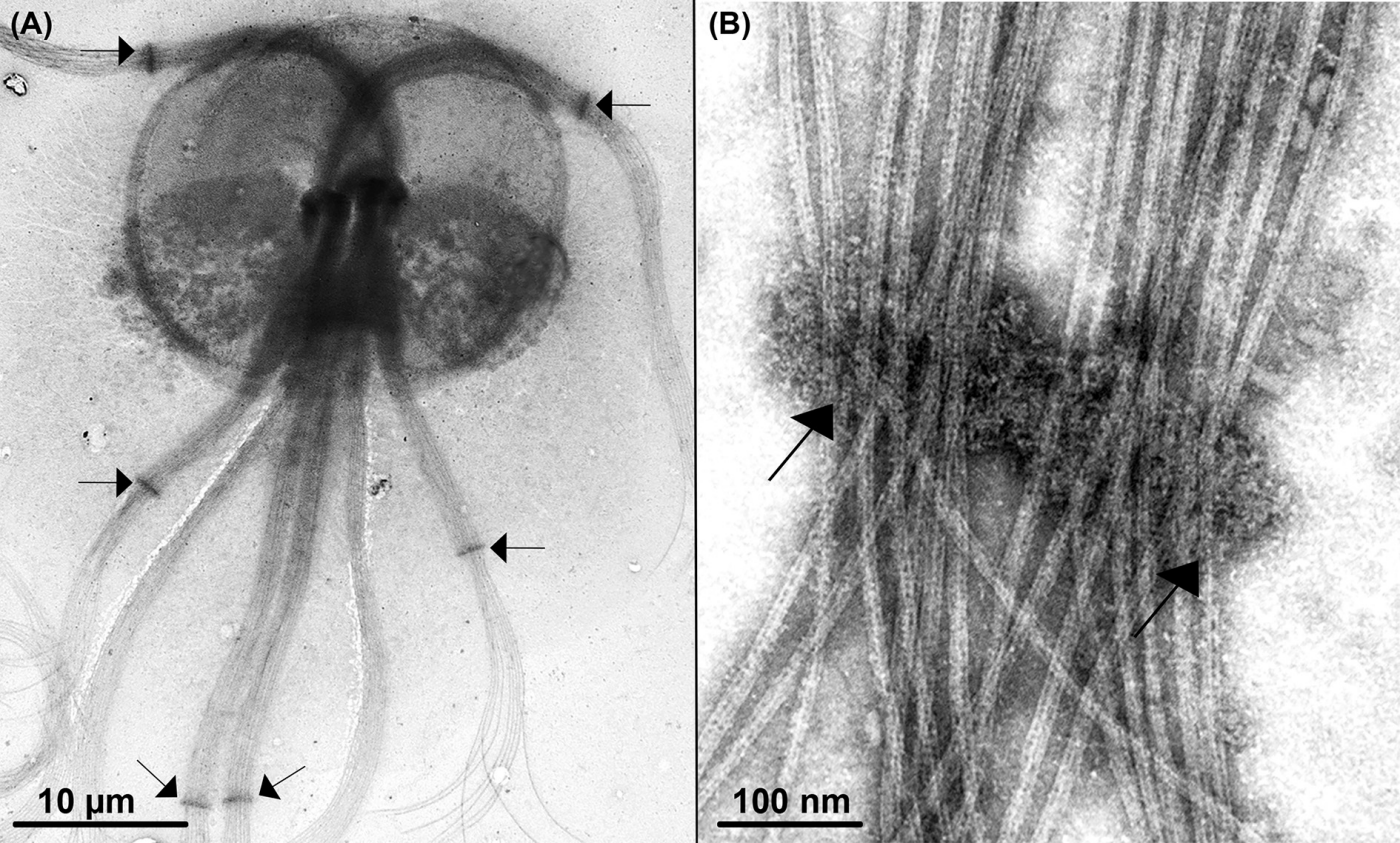
Negative staining of *Giardia intestinalis* trophozoites following membrane extraction (**A**) Extra-axonemal structures (arrows) are visible along the axonemes of the anterior, postero-lateral, and caudal flagella. (**B**) These structures form a belt-like arrangement surrounding the axonemes (arrows). ((Images from the authors [134].Elsevier permission for reuse.

*G. intestinalis* basal bodies are not localized close to the membrane, as seen in most other flagellated organisms. Instead, they are located deeper within the cell, between the two nuclei. Because of this organization, the axoneme still extends a considerable distance through the cytoplasm until it emerges as a free flagellum. The region where the axoneme exits the cell body is called the flagellar pore [129]. It has a distinct organization, characterized by a prominent surface forming a ring arrangement [134]. Detailed analysis of the flagellar externalization region reveals a specialized internal architecture. A notable structure associated with the outer doublets is composed of ribbon-like or globular aggregates that form a belt surrounding the axoneme, suggesting a potential role in anchoring or stabilizing the flagellum during its emergence [134] (Figure 22).

Associated with this intracytoplasmic portion of the axonemes are accessory structures whose functions, protein nature, and structural arrangement remain poorly understood. The axonemes of the anterior flagella have three associated structures, including: the fimbriae, structures characterized by light and dark bands in the proximal region of the axonemes [108,114]; the dense rod, a dense mass of fibers observed when both axonemes cross in the anterior portion of the cell; and the marginal plate, a boomerang-shaped structure connected to the upper part of the axoneme by small, flexible bridges [135]. Dense rods are also found next to the axoneme of the posterior flagella. The axonemes of the ventral flagella are connected to the paraxial rod by thin filaments [136]. The axoneme of the caudal flagellum is associated with the funis, forming the caudal complex.

**Figure 23:**
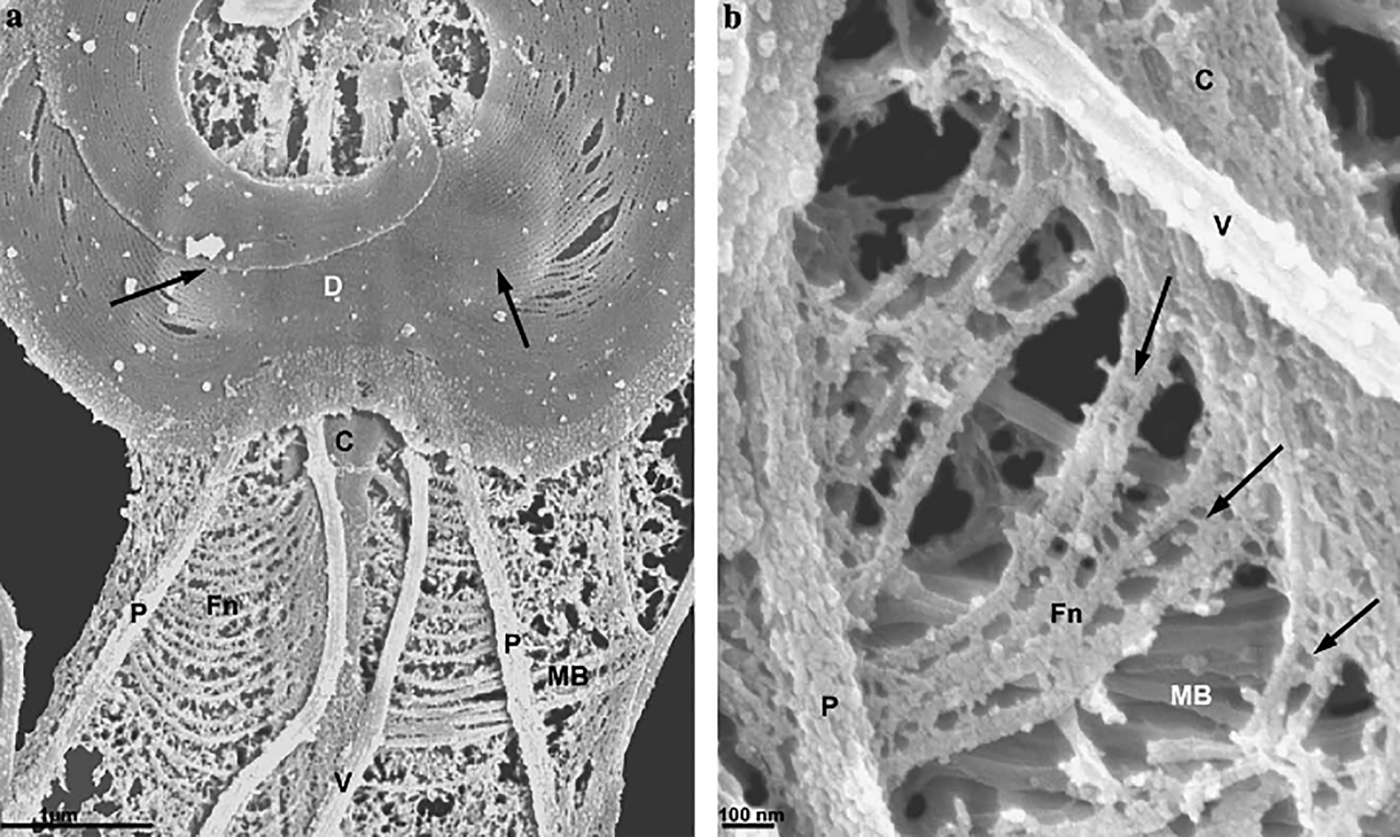
Negative staining of *Giardia intestinalis* trophozoites following membrane extraction (**A**) Microtubules of the funis (Fn) emanate from the caudal flagella (C) and anchor to the postero-lateral flagella (P). Arrows indicate impressions of the nuclei, which are positioned dorsally to the ventral adhesive disc (D). (**B**) Note the presence of connecting links between funis microtubules (arrows), many of which were disrupted by the extraction procedure. MB - median body. ((Images from the authors. Images from the authors with permission from Elsevier for reuse [137].

Among the accessory structures described above, the funis is the most extensively characterized. This structure is formed by two microtubular sheets that surround and extend dorsally and ventrally to the caudal axonemes. In the posterior region of the cell, some microtubules from each sheet disperse toward the posterior axonemes or the plasma membrane [137]. It has been suggested that the funis contributes to the dorso-lateral movement of the caudal region [137] (Figure 23).

As mentioned above, the basal bodies of *G. intestinalis* are localized between the two nuclei and are arranged in two tetrads. The basal bodies of the anterior flagella are oriented toward the anterior end of the cell, while those of the ventral, caudal, and posterolateral flagella are oriented posteriorly. The basal bodies of the anterior flagella display two distinguishing features: they are slightly inclined toward the nucleus and remain in contact with the basal bodies of the posteriorly oriented flagella [108].

The structure of the basal bodies of *G. intestinalis* was recently analyzed [108]. Two distinct domains have been identified: a cartwheel configuration at the proximal end and a rudimentary transition zone at the distal end, characterized by two plates that nucleate the central pair [108].

Proteomic analysis and immunofluorescence-based approaches using specific antibodies have identified several conserved basal body components in *G. intestinalis*, including tubulins, centrin, gamma-tubulin, calmodulin, alpha-giardins, and *Giardia*-specific proteins with no known homologues in other species [105,111,138]. The presence of centrin was also observed in fibrils that connect the basal bodies to the nucleus [139–141].

*G. intestinalis* also has components related to the regulation of the flagellar length, including intraflagellar transport (IFT) proteins, the BBsome complex, and motor proteins such as kinesin-2a/b and dynein. Using live-cell imaging with yellow-green fluorescent protein, it was possible to monitor the movement of some of these elements. For example, IFT particles and Kinesin 2a/2b accumulated in the flagellar pore region and moved between the pore and the flagellar tip [142,143]. Functional studies have demonstrated that the knockout of IFT88 and kinesin 2a/2b causes flagellar shortening, underscoring the critical role these proteins play in flagellar elongation [142,143]. As proposed by [142], flagellar assembly occurs via anterograde IFT, and it is independent of length. In contrast, flagellar disassembly is length-dependent and regulated by kinesin-13, a motor protein that acts at the distal end of the axoneme, causing the depolymerization of microtubules. These findings suggest that the balance between assembly via IFT and disassembly via kinesin-13 establishes and maintains the flagellar length [142]. Additional pathways may be involved in this regulation. Proteins such as polo-like kinase (PLK), cyclin-dependent kinase (CDK), NIMA-related kinases (NEKs), and end-binding protein 1 (EB1) have been implicated in this process. Knockdown of these proteins also causes defects in flagellar length, suggesting that they may regulate IFT in motor proteins [144,145]. Notably, PLK and CDK are also found in the flagellar pore region, further supporting their involvement in length regulation.

*Giardia* flagella are essential for parasite motility, with each pair exhibiting distinct beating frequencies and movement patterns. The anterior pair of flagella exhibits a helical beating pattern and may be involved in the rotational movement of the trophozoite when it is free in the medium [127,128]. The latero-posterior pair of the flagella creates a propulsive force that enables planar swimming, particularly when the parasite is near a substrate [127]. Ventral flagella present a sinusoidal beating parallel to the longitudinal axis of the cell [127,128]. This pair may be necessary to maintain the trophozoite adhesion to the host intestinal epithelial [126,127]. Interestingly, the caudal flagella do not show active external beating. However, the movement of its intracytoplasmic portion (caudal complex) may be responsible for the lateral bending and dorsal-ventral flexion of the trophozoite caudal region.

Flagellar motility also plays an important role in cell division. The beating of the internal axonemes of the caudal flagella generates the mechanical force necessary for daughter cell separation [146]. Given that myosin II is absent in *Giardia*, these findings strongly suggest that motility-based mechanisms are required for cytokinesis.

## Median body

**Figure 24:**
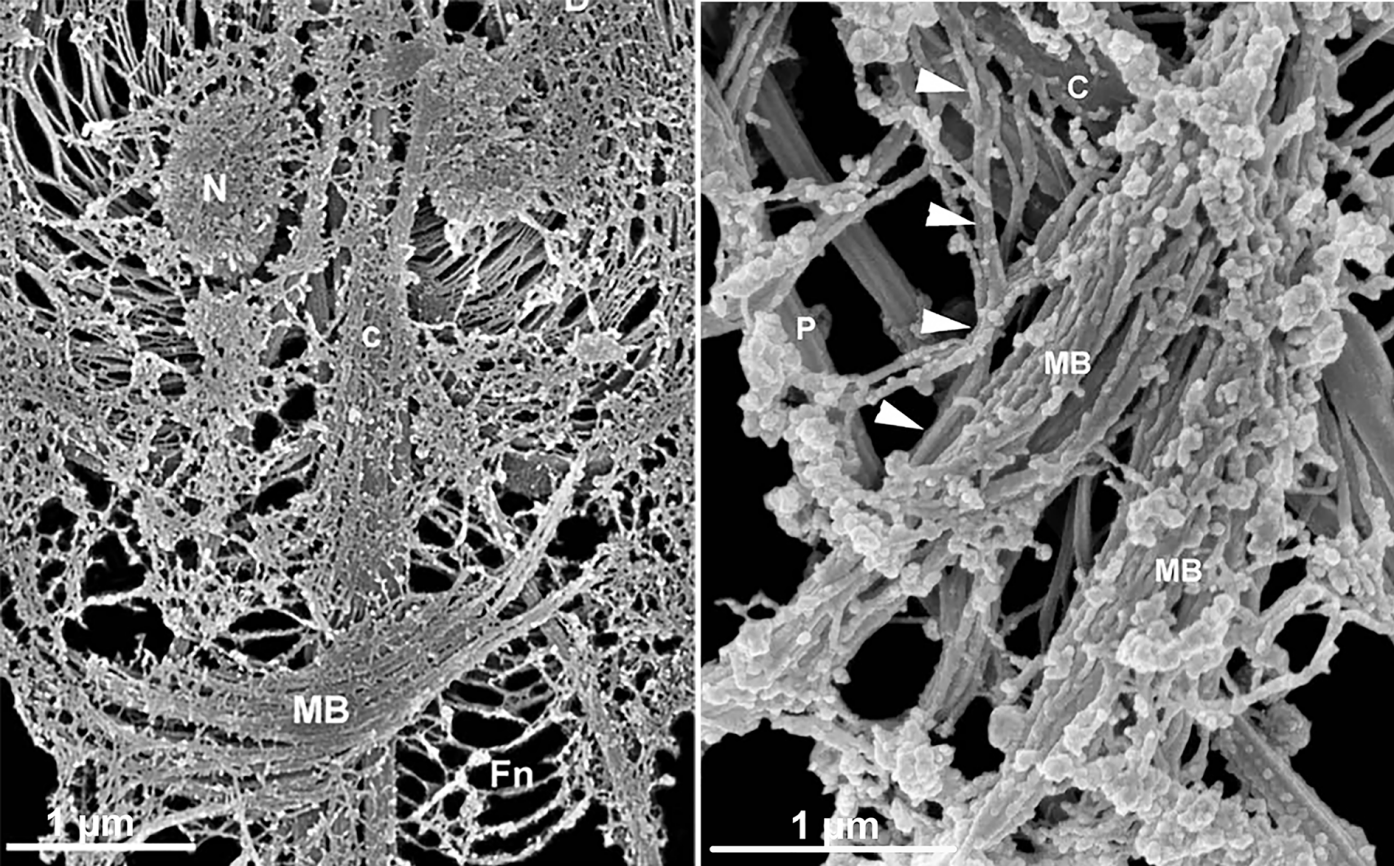
High-resolution SEM images of the median body cytoskeleton (**A**) The median body (MB) appears curved and oriented toward the cell's anterior region. (**B**) Individual microtubule fascicles that constitute the median body (MB) are clearly observed. N - Nucleus; C - Caudal flagella; P - Posterior-lateral flagella; Arrowheads - Funis microtubules. Images from the authors [147] with permission from Wiley Copyright Transfer Agreement (CTA).

The median body is used as a taxonomic parameter because its shape varies among *Giardia* species. In *G. intestinalis*, it typically exhibits a club-like shape and is positioned in the transverse midplane of the cell. High-resolution scanning electron microscopy of detergent-extracted cells has revealed detailed features of this structure [147]. It is composed of a variable number of fascicles, each containing a different number of microtubules (Figure 24). Its size ranges from 0.8 μm to 8 μm, a variation that may reflect differences in microtubule polymerization. Immunofluorescence analysis using antibodies specific for post-translational modifications detected monoglycylated, polyglycylated, acetylated, and tyrosinated tubulin within the median body [116], suggesting that the median body comprises a heterogeneous population of microtubules that contain both stable and dynamic forms. In addition to tubulin, other proteins, such as kinesin-13, polo-like kinase, end-binding 1 protein (EB-1), and a coiled-coil cytolinker protein, CLP259, have been identified [145,147–149].

The microtubules of the median body appear to be associated with several cellular structures, including the caudal axonemes, the funis, and the plasma membrane [147]. Although the precise function of these associations remains unclear, it has been proposed that the median body may contribute to the parasite’s caudal movements [147]. Furthermore, the detection of α-giardin—a protein characteristic of the microribbons that compose the ventral disc—in the median body has led to the hypothesis that this structure may serve as a reservoir of cytoskeletal microtubules or act as a template for assembling new disc structures [115,147]. The presence of γ-tubulin and centrin in the median body has also supported the idea that it may function as a microtubule-organizing center [139].

## Actin

**Figure 25:**
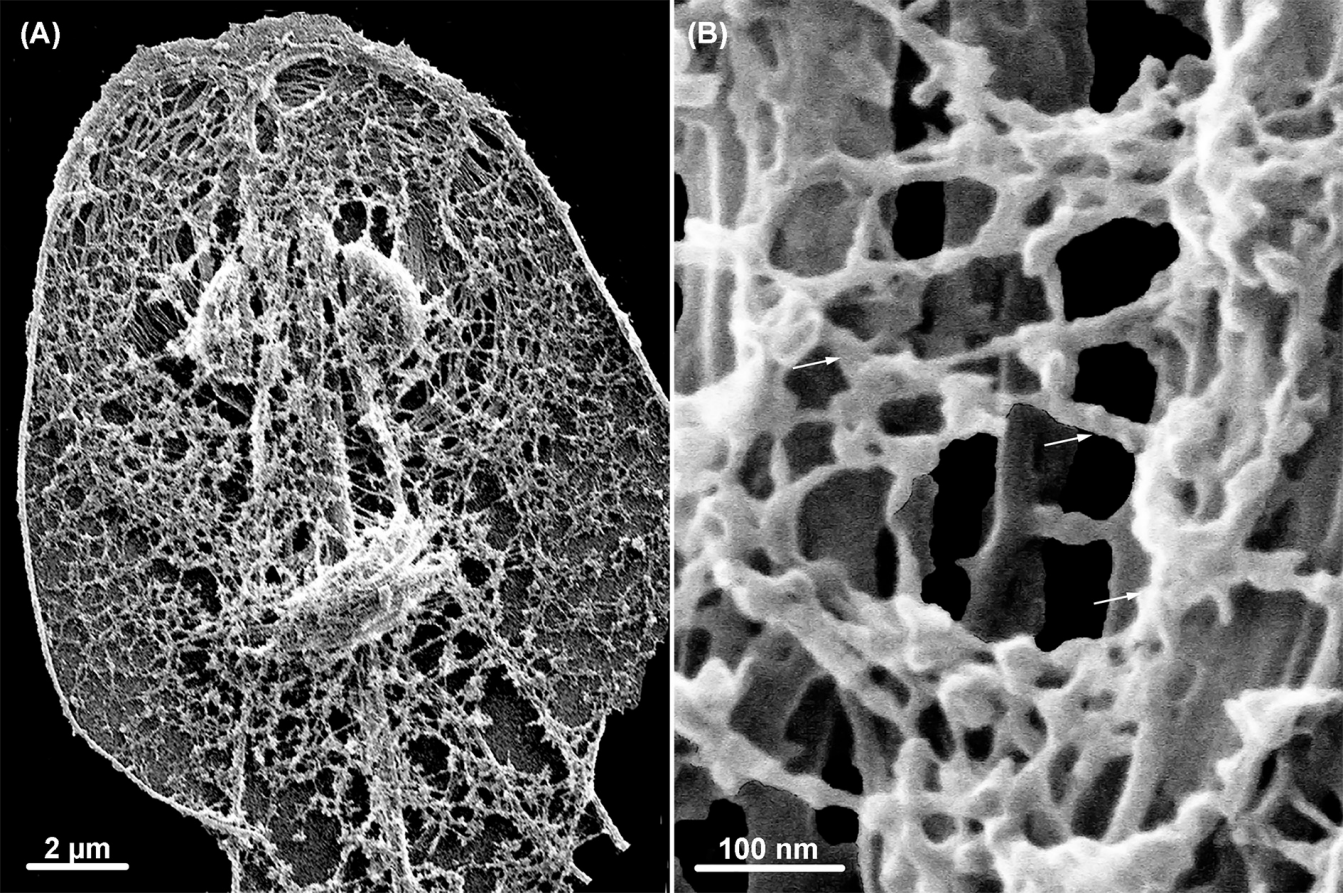
Visualization of the dorsal side of *G. intestinalis* after plasma membrane extraction (**A**) Cytoplasmic filaments extending throughout the entire body. (**B**) Higher magnification of (A), showing a filamentous network (arrows) (images from the authors [113]).

Although actin is a highly conserved cytoskeletal component in most eukaryotes, *G. intestinalis* contains only a single copy of the actin gene encoding a protein with 58% identity to human actin. Previous studies using fluorescence microscopy, combined with *Giardia*-specific anti-actin antibody or phalloidin staining, revealed that actin is distributed throughout the parasite, including positive labeling in the axonemes, ventral disc, median body, and near the nuclei [150]. The *in vitro* polymerization of Giardia actin was investigated, demonstrating its ability to form short filaments [150]. Later, using helium ion microscopy, high-resolution scanning electron microscopy, and immunogold labeling [113], characterized a network of filaments approximately 9 nm in diameter (Figure 25). These filaments, which reacted with anti-actin antibody, extended across the entire dorsal surface of the cell [113].

To gain insights into the function of *Giardia* actin, several studies have used actin-disrupting drugs and gene knockdown approaches targeting actin [150–152]. Under both conditions, alterations were observed in cell structure (flagella, caudal region), membrane trafficking, and cytokinesis in cells treated with cytochalasin or jasplakinolide, as well as in actin knockdown mutants. Furthermore, one group [153] showed that Rab11 participates in the actin cytoskeleton-mediated transport of encystation-specific vesicles (ESVs) to the cyst wall.

Although *G. intestinalis* lacks canonical actin-binding proteins, it has a conserved set of actin-interacting proteins [154]. One of these, Disc and Actin Associated Protein 1 (DAAP1), interacts with *Giardia* actin (GiActin) and localizes to the ventral disc [155]. Interestingly, depletion of either GiActin or DAAP1 impairs parasite adhesion, suggesting that actin dynamics play a critical role in maintaining disc structure and host attachment [155].

## The cytoskeleton of *Entamoeba histolytica*

*E. histolytica* is the most medically relevant representative among amoeba species, found in a wide variety of environments, especially aquatic ones. They are characterized by motility that involves the formation of pseudopods. Here, we will discuss only the species *E. histolytica*, which has a high incidence, resulting in approximately 40–50 million cases per year and approximately 50,000 deaths annually [156,157]. Its life cycle is relatively simple, involving a highly motile trophozoite and a slightly rounded cyst with a cyst wall.

**Figure 26:**
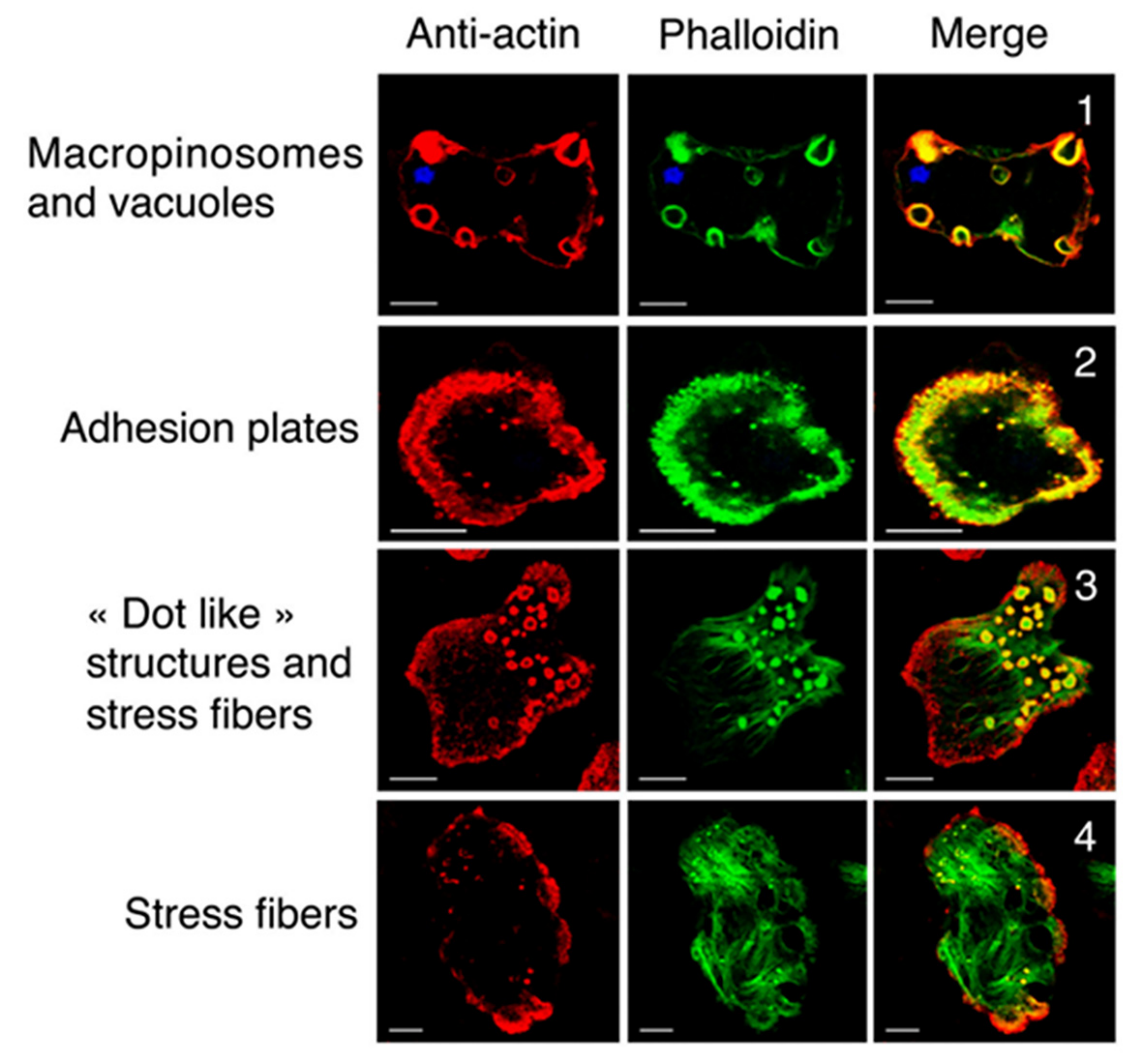
Cellular localization of G-actin and F-actin in *E. histolytica* Cellular localization of G-actin and F-actin in *E. histolytica* trophozoites using confocal microscopy in various focal planes (after [165]). Permission as Open Access from Frontiers in Cell Infection and Microbiology. This is an open-access image distributed under the terms of the Creative Commons Attribution License (CC BY).

The cytoskeleton of *E. histolytica* is relatively simple from a structural point of view, lacking structures formed by microtubules [158], except for the mitotic spindle, or intermediate filaments [159,160]. It is extremely rich in actin, which forms actin filaments involved in several cell motility processes, including reorganization at the cell periphery during cystogenesis and chitin polymerization [161], as well as in the formation of phagosomes, which is quite intense in this protist [162], where actin, Arp proteins, actin-binding proteins, PI3 kinase, activated protein kinase, and RHO GTPases are all involved in phagocytosis and signaling pathways in *E. histolytica* with the participation of actin. For instance, EhRho1 and EhGEF are recruited to endocytic sites [162]. Other relevant proteins have also been identified. Examples include formin, which stabilizes microfilaments, ABP-120 (filamin), which organizes microfilaments in orthogonal networks [163], profilin [160], coactosin, which stabilizes F-actin [163], vinculin, α-actnin, myosins, and others [164]. Previous authors [165] conducted a detailed analysis of *E. histolytica* actin using proteomics, protein structure modeling, and bioinformatics to compare *E. histolytica* actin with that of human cells. Most of these components were localized in the protist using confocal microscopy (Figure 26).

## Perspectives

Important advances have occurred in the last 10 years in the field of mammalian cell cytoskeleton, especially through biochemical and molecular approaches combined with cryoelectron tomography. In relation to pathogenic protists, significant advances have also been made with *T. brucei* and Apicomplexa, especially *Toxoplasma* and *Plasmodium*, where it is possible to obtain parasite strains that either express or lack specific genes. Only recently has it become possible to edit specific genes of *T. cruzi* using CRISPR–Cas9. However, gene interference in *Trichomonas* and *Giardia* remains challenging. We expect significant advances in the next few years through the use of cryo-electron tomography, particularly in characterizing the three-dimensional structures of some proteins *in situ* via sub-tomogram averaging.

## Data Availability

The data are available upon request.

## Abbreviations

CDK: cyclin-dependent kinase
DAAP1: Disc and Actin Associated Protein 1
DMT: doublet microtubule
EB1: end-binding protein 1
ESV: encystation-specific vesicle
ICMT: intraconoidal microtubule
NEK: NIMA-related kinase
PBF: parabasal filament
PLK: polo-like kinase
VPP: Vortex Phase Plates
